# ADSCs-Exo Attenuate NET Formation via the NADPH/MAPK Pathway and Mitigate NETs-Mediated Exacerbation of Hepatocyte Ferroptosis in a Miniature Pig Model of LIRI

**DOI:** 10.3390/cells15111040

**Published:** 2026-06-05

**Authors:** Xiangyu Lu, Pujun Li, Lei Cao, Tao Liu, Yajun Ma, Yue Wang, Chenxi Piao, Hongbin Wang

**Affiliations:** 1 College of Veterinary Medicine, Northeast Agricultural University, Harbin 150030, China; lxy1997202206@163.com (X.L.); pujunli1024@163.com (P.L.); caolei_001010@163.com (L.C.); liutaotiger@163.com (T.L.); mayajun1994@126.com (Y.M.); pcx7001@163.com (C.P.); 2 Heilongjiang Provincial Key Laboratory of Pathogenic Mechanism for Animal Disease and Comparative Medicine, Harbin 150030, China; 3 College of Animal Science and Technology, Henan University of Science and Technology, Luoyang 471000, China; wangyuemooney@163.com

**Keywords:** miniature pigs, liver ischemia-reperfusion injury, adipose-derived mesenchymal stem cell exosomes, neutrophil extracellular traps, ferroptosis

## Abstract

**Highlights:**

**What are the main findings?**

- NETs exacerbate hepatocyte ferroptosis in LIRI, with MPO identified as a key mediator of NET-induced hepatocellular injury.
- ADSCs-Exo suppress NET formation by inhibiting the NADPH/MAPK pathway, thereby alleviating hepatocyte ferroptosis and liver injury in a miniature pig model.

**What is the implication of the main finding?**

- ADSCs-Exo represent a promising cell-free therapeutic strategy for reducing liver ischemia–reperfusion injury during liver transplantation and hepatectomy.
- The miniature pig LIRI model provides a clinically relevant large-animal platform for improving the translational value of liver injury research.

**Abstract:**

The link between neutrophil extracellular traps (NETs) and hepatocyte ferroptosis in liver ischemia–reperfusion injury (LIRI) is unclear. Adipose-derived mesenchymal stem cell exosomes (ADSCs-Exo) hold therapeutic potential for LIRI. This study employed miniature pigs to investigate the NETs’ role and ADSCs-Exo’s protection in LIRI. In vitro, established hepatocyte oxygen-glucose deprivation/reoxygenation (OGD/R) model and Transwell co-culture system with polymorphonuclear neutrophils (PMNs). In vivo, a laparoscopic minimally invasive LIRI model was constructed in miniature pigs, followed by ADSCs-Exo intervention. Results demonstrated that NETs exacerbate OGD/R-induced hepatocyte ferroptosis via myeloperoxidase. ADSCs-Exo inhibited NET formation via the NADPH/MAPK pathway, thereby mitigating ferroptosis, and ultimately improved liver histopathology and function. This study is the first to demonstrate in a large animal model that ADSCs-Exo alleviate LIRI by inhibiting NET formation via the NADPH/MAPK pathway, consequently attenuating hepatocyte ferroptosis. These findings provide novel insights into LIRI pathogenesis, support the translational potential of ADSCs-Exo as a cell-free therapeutic strategy, and highlight the value of the miniature pig model in liver research.

## 1. Introduction

Liver ischemia–reperfusion injury (LIRI) is a major clinical complication associated with liver transplantation, hepatectomy, and abdominal trauma. It frequently causes severe hepatic structural damage and functional impairment and may ultimately lead to graft failure [1]. The pathogenesis of LIRI is complex and multifactorial, involving the dysregulation of energy metabolism [2], oxidative stress [3], and inflammatory cascades [4]. Owing to this pathological complexity, therapeutic strategies targeting a single pathway often show limited efficacy. Therefore, a comprehensive understanding of the mechanisms underlying LIRI and the development of effective therapeutic interventions are essential for preventing hepatic injury, improving the success rate of liver transplantation, reducing postoperative complications, and promoting the safe use of marginal donor livers. Such advances are of considerable significance for both basic research and the clinical management of liver diseases.

Accumulating evidence indicates that LIRI represents an endogenous, localized innate immune-inflammatory response triggered by ischemia-hypoxia stress, characterized by Kupffer cell activation and polymorphonuclear neutrophil (PMN) recruitment [5]. As the most abundant immune cells in the circulation, PMNs play a central role in inflammatory responses. Once recruited to sites of injury, PMNs aggravate hepatocellular damage, and the extent of their infiltration has been recognized as an important indicator of LIRI severity [6]. PMNs are among the first immune cells to arrive at ischemic tissues after blood flow restoration. In the inflammatory microenvironment, they participate in host defense through phagocytosis, reactive oxygen species (ROS) generation, degranulation, and the release of neutrophil extracellular traps (NETs) [7,8,9,10]. However, increasing evidence suggests that this protective response may become dysregulated under pathological conditions. Excessive NET formation has been shown to contribute to tissue injury and further amplify inflammatory damage [11]. Consistently, NETs have been implicated in the progression of ischemia–reperfusion injury in multiple organs and tissues, including the myocardium [12], kidneys [13], brain [14] and liver [15].

Ferroptosis is a form of regulated cell death characterized by iron dependence and lipid peroxidation [16]. Previous studies have demonstrated that ferroptosis plays an important role in liver diseases [17] and regulates hepatic injury, inflammation, and oxidative stress [18]. In a mouse model of LIRI, ferrostatin-1 (Fer-1), a specific inhibitor of ferroptosis, was shown to reduce the expression of inflammatory factors and attenuate the infiltration of PMNs and macrophages [19]. These findings suggest a close association between neutrophil-mediated inflammatory responses and ferroptosis during LIRI. Although studies in other ischemia–reperfusion injury models have demonstrated that NETs exacerbate tissue damage through multiple mechanisms, the relationship between NET formation and ferroptosis in LIRI remains unclear.

Adipose-derived mesenchymal stem cell-derived exosomes (ADSCs-Exo) are nanoscale extracellular vesicles with important paracrine functions. They are considered key mediators of the biological activities of adipose-derived mesenchymal stem cells (ADSCs), including tissue repair, immunomodulation, and anti-inflammatory regulation. Accordingly, ADSCs-Exo have been widely investigated in regenerative medicine and disease treatment. In the context of LIRI, ADSCs-Exo have been reported to alleviate hepatic injury by transferring bioactive molecules, including proteins, nucleic acids, and lipids. Their protective effects are mainly associated with the inhibition of oxidative stress [20], inflammatory responses [21], and endoplasmic reticulum stress [22], while promoting tissue regeneration [23]. Nevertheless, whether ADSCs-Exo regulate NET formation and NETs-mediated hepatocellular injury remains largely unknown.

Although mice and rats are widely used as experimental models because of their ease of handling, relatively low cost, and short experimental cycles [24,25], their physiological and anatomical differences from humans limit the clinical translation of findings obtained from these models. In contrast, miniature pigs share greater similarities with humans in terms of body size, physiological and biochemical characteristics, and anatomical structure [26]. Together with relatively fewer ethical concerns [27], these features make miniature pigs valuable models for comparative medicine and translational research.

Based on these considerations, the present study established an in vitro co-culture system using hepatocytes subjected to oxygen-glucose deprivation/reoxygenation (OGD/R) and PMNs, as well as an in vivo miniature pig model of LIRI, to investigate the protective effects and underlying mechanisms of ADSCs-Exo. Using these models, we explored whether ADSCs-Exo inhibit NET formation through the NADPH/MAPK signaling pathway and thereby alleviate hepatocyte ferroptosis. From the perspectives of veterinary medicine and comparative medicine, this study aims to provide experimental and theoretical evidence for elucidating the pathogenesis of LIRI and the protective mechanisms of ADSCs-Exo, thereby supporting the translational development of therapeutic strategies for liver diseases.

## 2. Materials and Methods

### 2.1. Animals

Thirty healthy Bama miniature pigs aged 4–6 months and weighing 20–25 kg were included in this study. The animals were maintained under standard housing conditions with free access to food and water throughout the experimental period. No animals were euthanized at the experimental endpoint. After completion of the experimental procedures, all pigs were returned to routine housing and continuously maintained under normal husbandry conditions.

### 2.2. Preparation of ADSCs-Exo

The isolation of ADSCs and ADSCs-derived exosomes (ADSCs-Exo) was performed according to a previously described protocol with minor modifications [28]. Briefly, inguinal adipose tissue was harvested from miniature pigs, minced into small pieces, and digested with 0.1% type I collagenase (BioFroxx, Einhausen, Germany). The isolated ADSCs were cultured in low-glucose DMEM (Gibco, Waltham, MA, USA) supplemented with 10% FBS (HyClone, Lgan, UT, USA), 2 mM L-glutamine, and 100 U/mL penicillin-streptomycin. The cells were maintained at 37 °C in a humidified incubator containing 5% CO_2_ (BB150, Thermo Fisher, Waltham, MA, USA). The culture medium was replaced every 48 h, and cells were passaged at a ratio of 1:2 to 1:3 when they reached 80–90% confluence.

Cell growth was observed daily under an optical microscope. ADSCs at passages 3–5 were harvested and prepared as single-cell suspensions for phenotypic identification. The cells were incubated with antibodies against CD29, CD44, CD90, and CD11b. The proportion of positive cells was detected by flow cytometry, and the data were analyzed using FACSDiva software (version 6.1.3; BD Biosciences, New York, NY, USA). In addition, adipogenic and osteogenic differentiation of ADSCs was induced using commercial differentiation kits (OriCell, Guangzhou, China) according to the manufacturer’s instructions. The antibodies used for ADSCs identification are listed in Table 1.

ADSCs-Exo were isolated using a combination of ultrafiltration and differential ultracentrifugation, as previously described with modifications [29,30]. Briefly, ADSCs at passages 3–5 were serum-starved for 24–36 h, after which the conditioned medium was collected. The medium was sequentially centrifuged at 300× *g* for 15 min to remove viable cells, 2000× *g* for 25 min to remove dead cells, and 10,000× *g* for 50 min to eliminate cellular debris. The resulting supernatant was filtered through a 0.22-μm sterile filter (Merck Millipore, Burlington, MA, USA) and concentrated using a 100-kDa molecular weight cutoff ultrafiltration tube (Merck Millipore, Burlington, MA, USA) at 2500 rpm for 10 min. The concentrate was then ultracentrifuged at 100,000× *g* for 2 h using a Ti 50.2 rotor (Beckman Coulter, Brea, CA, USA). The final exosome pellet was resuspended in ice-cold sterile PBS and stored at −80 °C until further use.

The size distribution and concentration of ADSCs-Exo were determined by nanoparticle tracking analysis (NTA; Particle Metrix, Inning am Ammersee, Germany). The morphology of ADSCs-Exo was examined by transmission electron microscopy (TEM; Hitachi, Japan) after negative staining with phosphotungstic acid. The characteristic exosomal markers CD63, CD81, and TSG101 were detected by Western blotting. The antibodies used for ADSCs-Exo identification are listed in Table 1.

### 2.3. Isolation, Culture and Identification of Hepatocytes

Primary hepatocytes were isolated from neonatal piglets within 1 week of birth using a two-step perfusion method [31]. The isolated cells were seeded and cultured for 4 h in Medium A, consisting of high-glucose DMEM (HyClone, Lgan, UT, USA), 10% fetal bovine serum (FBS), 2 mM L-glutamine, 100 U/mL penicillin, 125 U/L insulin, and 0.2 μg/mL dexamethasone. After cell attachment, the cultures were washed with phosphate-buffered saline (PBS) and maintained in serum-free Medium B, which was identical to Medium A except for the absence of FBS.

Hepatocyte morphology and proliferation were observed under an inverted optical microscope. For glycogen staining, cells were fixed with 4% paraformaldehyde and stained using a periodic acid–Schiff (PAS) glycogen staining kit (Solarbio, Beijing, China) according to the manufacturer’s instructions. For immunofluorescence staining, cells were fixed with 4% paraformaldehyde, permeabilized with 0.1% Triton X-100, blocked with 3% BSA, and incubated overnight at 4 °C with an anti-CK-18 antibody (Cat# 10830-1-AP, Proteintech, Wuhan, China). The cells were then incubated with an FITC-conjugated secondary antibody (550036, Zenbio, Chengdu, China) for 1 h at room temperature. After mounting medium was applied, the cells were observed under a fluorescence microscope at an excitation wavelength of 495 nm and an emission wavelength of 519 nm.

### 2.4. Isolation, Cultivation and Identification of PMNs

Peripheral blood neutrophils were isolated from anticoagulated pig blood using a porcine peripheral blood neutrophil isolation kit (Solarbio, Beijing, China) according to the manufacturer’s protocol. Briefly, reagents A and C were sequentially added to the anticoagulated blood, and the samples were centrifuged to separate the cellular components. The white cell layer containing neutrophils was carefully transferred to a new tube, washed, and resuspended in RPMI 1640 medium supplemented with FBS (HyClone, Lgan, UT, USA), L-glutamine (Biosharp, Hefei, China), and penicillin-streptomycin (Beyotime Biotechnology, Shanghai, China).

A smear was prepared using 10 μL of neutrophil suspension, air-dried, and stained with Wright–Giemsa stain (Base, Shanghai, China). Briefly, solution A was added to the smear and incubated for 1 min, after which solution B was added and gently mixed. The smear was stained for 4–10 min, rinsed with water, air-dried, and then examined under a light microscope.

### 2.5. Hepatocyte Uptake of ADSCs-Exo

Hepatocytes were seeded onto cell coverslips and cultured until they reached the appropriate confluence. ADSCs-Exo were pre-labeled with Aco600 fluorescent dye (Umibio, Shanghai, China) and resuspended in serum-free medium at the optimal working concentration. The labeled ADSCs-Exo suspension was then added to the hepatocyte culture system, and the cells were incubated at 37 °C in a humidified incubator containing 5% CO_2_ for 24 h in the dark.

After incubation, the exosome-containing medium was removed, and the hepatocytes were washed three times with pre-warmed PBS for 5 min each to remove unbound exosomes. Subsequently, DiO working solution was added to label the cell membrane, and the cells were incubated at 37 °C for 8 min in the dark. After DiO staining, the cells were washed three times with PBS for 5 min each to remove excess dye and then fixed with 4% paraformaldehyde at room temperature for 10 min.

The coverslips were carefully removed from the culture plates and mounted with DAPI-containing antifade mounting medium to stain nuclei and prevent fluorescence quenching. Finally, the labeled hepatocytes were observed under a laser scanning confocal microscope (LSCM), and representative images were captured to evaluate the uptake of ADSCs-Exo by hepatocytes.

### 2.6. Hepatocyte OGD/R Model

To establish an in vitro model of LIRI, hepatocytes were subjected to oxygen-glucose deprivation/reoxygenation (OGD/R). Briefly, cells were exposed to OGD by incubation in glucose-free Dulbecco’s phosphate-buffered saline (D-PBS) supplemented with 2 mM L-glutamine and 100 U/mL penicillin. The cells were then placed in a hypoxic chamber containing 5% CO_2_ and 95% N_2_ at 37 °C for 2 h. For reoxygenation, the OGD medium was replaced with Medium B, and the cells were returned to a normoxic incubator containing 5% CO_2_ at 37 °C for 6 h.

Pretreatments were applied 1 h before the OGD phase. Cells in the Fer-1 group were treated with 60 nM ferrostatin-1, whereas cells in the Exo group were treated with 10 μg/mL ADSCs-Exo. At the onset of reoxygenation, neutrophils were added to the upper chamber of a Transwell system at a density of 1 × 10^5^ cells/mL to establish the co-culture (Co) model. In the Co + Exo group, an additional 10 μg/mL ADSCs-Exo was added at the beginning of reoxygenation.

### 2.7. OGD/R Culture Medium Induces NETs

To investigate the effects of OGD/R-treated hepatocytes on neutrophils, conditioned medium was collected from hepatocytes subjected to OGD/R and used to stimulate neutrophils. Briefly, neutrophils were initially cultured in normal medium and then incubated with OGD/R-induced hepatocyte-conditioned medium (OGD/R-CM) for 6 h. For the DPI and Exo groups, neutrophils were pretreated with 5 nM DPI or 10 μg/mL ADSCs-Exo, respectively, for 1 h before OGD/R-CM stimulation.

### 2.8. Miniature Pig LIRI Model

Thirty healthy Bama miniature pigs, including equal numbers of males and females, were randomly assigned to five groups (*n* = 6 per group): sham operation group (Sham), ischemia–reperfusion injury group (IRI), ADSCs-Exo treatment group (Exo), clodronate liposome-mediated Kupffer cell depletion group (CI), and clodronate liposome-mediated Kupffer cell depletion combined with ADSCs-Exo treatment group (CE). A miniature pig model of LIRI combined with partial hepatectomy was established as previously described [32]. Briefly, the pigs were anesthetized by intravenous injection of Zoletil 100 at a dose of 1 mg/kg via the marginal ear vein. After endotracheal intubation, anesthesia was maintained with 2–3% isoflurane under mechanical ventilation with an oxygen flow rate of 1 L/min. A four-trocar surgical approach was established under a pneumoperitoneum pressure of 10 mmHg. The right hepatic lobe was subjected to 60 min of ischemia, followed by resection of the left hepatic lobe. In the Sham group, only abdominal exploration was performed under the same surgical conditions, without vascular occlusion or hepatectomy.

The treatments were administered as follows. Pigs in the Sham and IRI groups received 5 mL of PBS via the hepatic portal vein. Pigs in the Exo group received ADSCs-Exo at a dose of 200 μg per pig suspended in 5 mL of PBS via the hepatic portal vein immediately after hepatectomy. Pigs in the CI group were pretreated with clodronate disodium liposomes at a dose of 2.5 mg/kg via the marginal ear vein 24 h before surgery and then received 5 mL of PBS via portal vein injection after hepatectomy. Pigs in the CE group received the same clodronate liposome pretreatment 24 h before surgery, followed by portal vein injection of ADSCs-Exo at a dose of 200 μg per pig suspended in 5 mL of PBS after hepatectomy.

Blood samples were collected from the Cranial vena cava before surgery and on postoperative days 1, 3, and 7. Liver tissue samples were harvested at the same time points using laparoscopic minimally invasive techniques.

### 2.9. Cell Viability and Cytotoxicity

Cells were seeded into 96-well plates, and cell viability was assessed using a Cell Counting Kit-8 (CCK-8; MA0225, Meilunbio, Dalian, China) according to the manufacturer’s instructions. The absorbance was measured at 450 nm using an Epoch full-wavelength microplate reader (BioTek, Winooski, VT, USA).

Cell culture supernatants were collected from hepatocytes, and lactate dehydrogenase (LDH) release was measured using an LDH assay kit (Beyotime Biotechnology, Shanghai, China). The absorbance was measured at 490 nm using the same microplate reader.

### 2.10. Liver Function Analysis

Blood samples were allowed to stand at room temperature for 30 min and then centrifuged at 3500 rpm for 10 min to obtain serum. Serum levels of aspartate aminotransferase (AST), lactate dehydrogenase (LDH), alkaline phosphatase (ALP), and total bilirubin (TBIL) were measured using an automatic biochemical analyzer (TBA-2000FR, Canon Medical Systems Corporation, Tochigi, Japan).

Cell culture supernatants were collected and centrifuged at 400× *g* for 5 min to remove cellular debris. The supernatants were then used to measure alanine aminotransferase (ALT) and AST activities using commercial assay kits according to the manufacturer’s instructions (Nanjing Jiancheng Bioengineering Institute, Nanjing, China).

### 2.11. Measurement of Oxidative Stress

Liver tissues were homogenized in nine volumes of ice-cold normal saline to prepare 10% tissue homogenates. The homogenates were centrifuged at 4000 rpm for 15 min, and the supernatants were collected for subsequent analysis. The levels of superoxide dismutase (SOD), catalase (CAT), malondialdehyde (MDA), and glutathione (GSH) in liver tissue homogenates were measured using commercial assay kits according to the manufacturer’s instructions (Nanjing Jiancheng Bioengineering Institute, Nanjing, China).

For in vitro experiments, hepatocytes were collected and lysed to obtain cell lysates. The levels of SOD, CAT, MDA, and GSH in hepatocyte lysates were measured using the corresponding commercial assay kits according to the manufacturer’s instructions.

### 2.12. DCFH-DA Analysis

Liver tissues stored at −80 °C were embedded in optimal cutting temperature (OCT) compound and sectioned into 8-μm-thick frozen slices. The sections were incubated with dihydroethidium (DHE) at 37 °C for 30 min in the dark to detect ROS production in liver tissues. After incubation, the sections were washed with PBS and observed under a fluorescence microscope.

For in vitro ROS detection, hepatocytes and neutrophils were incubated with DCFH-DA for 20 min in the dark. After staining, the cells were washed to remove excess fluorescent probe and observed under a fluorescence microscope at an excitation wavelength of 488 nm and an emission wavelength of 525 nm.

### 2.13. Fe^2+^ Content Detection

Intracellular Fe^2+^ levels in hepatocytes were detected using an Fe^2+^ fluorescent assay kit (MeilunBio, Dalian, China) according to the manufacturer’s instructions. Briefly, hepatocytes were seeded into 24-well plates and treated according to the experimental design. At the end of the experiment, the culture medium was removed, and the cells were washed two to three times with staining buffer. An appropriate volume of staining working solution was then added, and the cells were incubated at 37 °C in a humidified incubator containing 5% CO_2_ for 30 min. After incubation, the cells were observed and photographed under a fluorescence microscope at an excitation wavelength of 543 nm and an emission wavelength of 580 nm.

### 2.14. ELISA Analysis

Liver tissues were homogenized to prepare 10% tissue homogenates, and the supernatants were collected for ELISA analysis. The levels of 4-hydroxynonenal (4-HNE), AA, CXCL1, CXCL2, CXCR2, ICAM-1, JAM-A, LFA-1, NE, dsDNA, and H3Cit in liver tissue homogenates were measured using commercial ELISA kits according to the manufacturer’s instructions (mmbio, Yancheng, China).

Serum samples were collected from each group, and the concentrations of IL-1β, IL-6, TNF-α, and IL-10 were determined using commercial ELISA kits according to the manufacturer’s instructions (mmbio, China).

### 2.15. MPO Immunofluorescence Staining

Liver tissue sections were deparaffinized, rehydrated, subjected to antigen retrieval, and blocked with 3% BSA. The sections were incubated overnight at 4 °C with an anti-MPO antibody (22225-1-AP, Proteintech, China). After washing with PBS, the sections were incubated with an FITC-conjugated secondary antibody (bs-0295G-FITC, Bioss, Beijing, China) for 1 h at room temperature in the dark. The nuclei were counterstained with DAPI (Beyotime, China), and the sections were mounted and observed under a fluorescence microscope at the appropriate excitation and emission wavelengths.

### 2.16. RT-qPCR

Total RNA was extracted from liver tissues using TRIzol reagent and quantified. The extracted RNA was reverse-transcribed into cDNA using a reverse transcription kit according to the manufacturer’s instructions (CWBIO, Taizhou, China). Real-time quantitative PCR (RT-qPCR) was performed using SYBR Green I fluorescent dye (CWBIO, China) on a LightCycler 480 system (Roche, Basel, Switzerland) with a three-step amplification protocol. The relative mRNA expression levels of target genes were calculated using the 2^^−ΔΔCt^ method, with β-actin used as the internal reference gene. The primers were synthesized by UW Genetics Technology, and the primer sequences are listed in Table 2.

### 2.17. Western Blotting

Cell samples were lysed with RIPA lysis buffer (MeilunBio, Dalian, China) supplemented with protease inhibitors (Beyotime, Shanghai, China) and phosphatase inhibitors (Proteintech, Wuhan, China). Protein concentrations were determined using a BCA protein assay kit (Beyotime, Shanghai, China). The protein samples were adjusted to equal concentrations with saline and loading buffer, boiled for denaturation, separated by SDS-PAGE (Epizyme, Cambridge, MA, USA), and transferred onto nitrocellulose (NC; Cytiva, Sweden) or polyvinylidene fluoride (PVDF; Merck Millipore, Germany) membranes.

The membranes were blocked with PVP4000 (Biosharp, Beijing, China) and incubated overnight at 4 °C with the corresponding primary antibodies. After washing, the membranes were incubated with secondary antibodies. Protein bands were visualized using an enhanced chemiluminescence (ECL) reagent (MeilunBio, Dalian, China) and detected with a Tanon 5200 imaging system. The optical density of each band was quantified using ImageJ software (Java 1.8.0_112, 64-bit). The antibodies used for Western blotting are listed in Table 3.

### 2.18. Statistical Analysis

Statistical analyses were performed using SPSS software (version 22.0). All data are presented as the mean ± standard deviation (SD). Differences among multiple groups were analyzed using one-way analysis of variance (ANOVA), followed by the least significant difference (LSD) post hoc test when appropriate. A value of *p* < 0.05 was considered statistically significant.

## 3. Results

### 3.1. ADSCs-Exo Alleviates Ferroptosis Exacerbation in Hepatocytes Induced by PMNs Co-Culture After OGD/R

ADSCs, ADSCs-Exo, hepatocyte, and PMNs were comprehensively characterized. The results confirmed that all cell types and ADSCs-Exo met the established identification criteria and exhibited high purity. Detailed characterization data are provided in Supplementary Figures S1 and S2. In addition, the results shown in Figure S3 demonstrated that hepatocytes efficiently internalized ADSCs-Exo.

**Figure 1:**
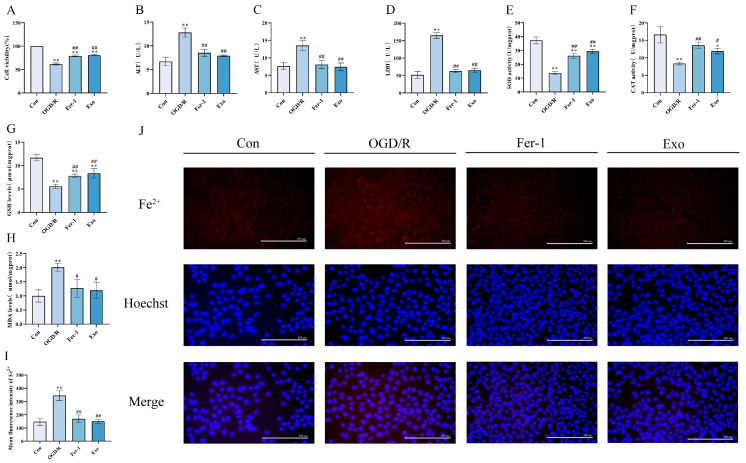
ADSCs-Exo attenuates hepatocyte ferroptosis following OGD/R. (**A**) Hepatocyte viability assay results in each group; (**B**–**D**) Hepatocyte function assay results in each group; (**E**) SOD assay results in each group of hepatocytes; (**F**) CAT assay results in each group of hepatocytes; (**G**) GSH assay results in each group of hepatocytes; (**H**) MDA assay results in each group of hepatocytes; (**I**) The average fluorescence analysis of intracellular Fe^2+^ staining in each group of hepatocytes; (**J**) Fe^2+^ staining results of intracellular in each group of hepatocytes (scale bar = 200 μm). Data were presented as mean ± SD. * 0.01 < *p* < 0.05, ** *p* < 0.01, versus the Con group. # 0.01 < *p* < 0.05, ## *p* < 0.01, versus the GOD/R group. *n* = 3.

Subsequently, an in vitro OGD/R model was established in hepatocytes to evaluate ischemia–reperfusion-induced injury and the protective effects of ADSCs-Exo. OGD/R exposure significantly reduced hepatocyte viability (Figure 1A, *p* < 0.01) and induced hepatocellular dysfunction, as evidenced by marked increases in ALT (Figure 1B), AST (Figure 1C), and l LDH (Figure 1D) release levels (all *p* < 0.01). OGD/R-induced injury was accompanied by impaired antioxidant capacity, as reflected by significant changes in antioxidant-related indicators (Figure 1E,F; *p* < 0.01), indicating the occurrence of oxidative stress. Moreover, intracellular Fe^2+^ levels were significantly increased after OGD/R exposure (Figure 1I,J; *p* < 0.01). This increase was accompanied by decreased GSH content and increased MDA content (Figure 1G,H; *p* < 0.01), suggesting enhanced lipid peroxidation and ferroptotic injury. Notably, pretreatment with either 60 nM Fer-1 (Figure S4A) or ADSCs-Exo similarly ameliorated these OGD/R-induced abnormalities (0.01 < *p* < 0.05), indicating that ADSCs-Exo alleviated OGD/R-induced hepatocyte ferroptosis.

As the predominant immune cells in the circulatory system, PMNs are preferentially recruited to injured sites after liver reperfusion and are widely recognized as key mediators of the post-reperfusion injury cascade [33]. Accordingly, in this study, PMNs were seeded into the upper chambers of Transwell inserts immediately after hepatocyte reoxygenation to establish a co-culture system with hepatocytes in the lower chambers. ADSCs-Exo were used as an intervention to investigate the damaging effects of PMNs on hepatocytes and the protective role of ADSCs-Exo.

**Figure 2:**
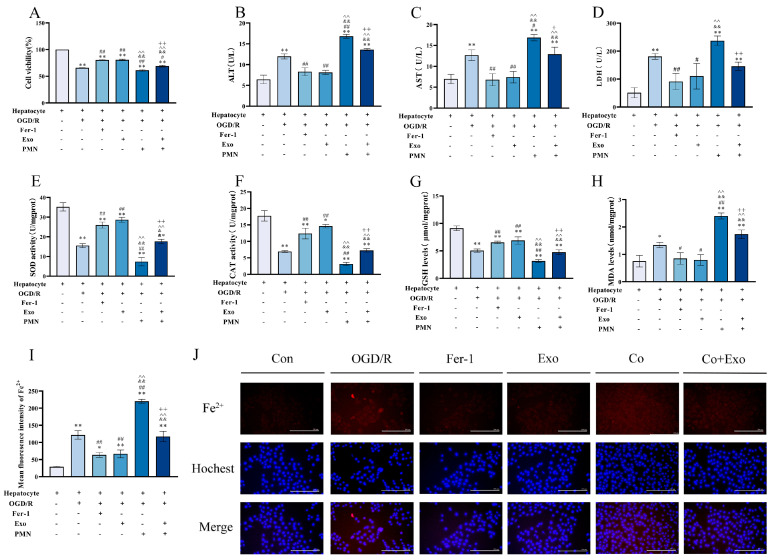
ADSCs-Exo alleviates ferroptosis exacerbation in hepatocytes induced by PMNs co-culture following OGD/R. (**A**) Hepatocyte viability assay results in each group; (**B**–**D**) Hepatocyte function assay results in each group; (**E**) SOD assay results in each group of hepatocytes; (**F**) CAT assay results in each group of hepatocytes; (**G**) GSH assay results in each group of hepatocytes; (**H**) MDA assay results in each group of hepatocytes; (**I**) The average fluorescence analysis of intracellular Fe^2+^ staining in each group of hepatocytes; (**J**) Fe^2+^ staining results of intracellular in each group of hepatocytes (scale bar = 200 μm). Data were presented as mean ± SD. * 0.01 < *p* < 0.05, ** *p* < 0.01, versus the Con group. # indicates 0.01 < *p* < 0.05, ## indicates *p* < 0.01, versus the OGD/R group. && *p* < 0.01, versus the Fer-1 group. ^^ indicates *p* < 0.01, versus the Exo group. + 0.01 < *p* < 0.05, ++ *p* < 0.01, versus the Co group. *n* = 3.

**Figure 3:**
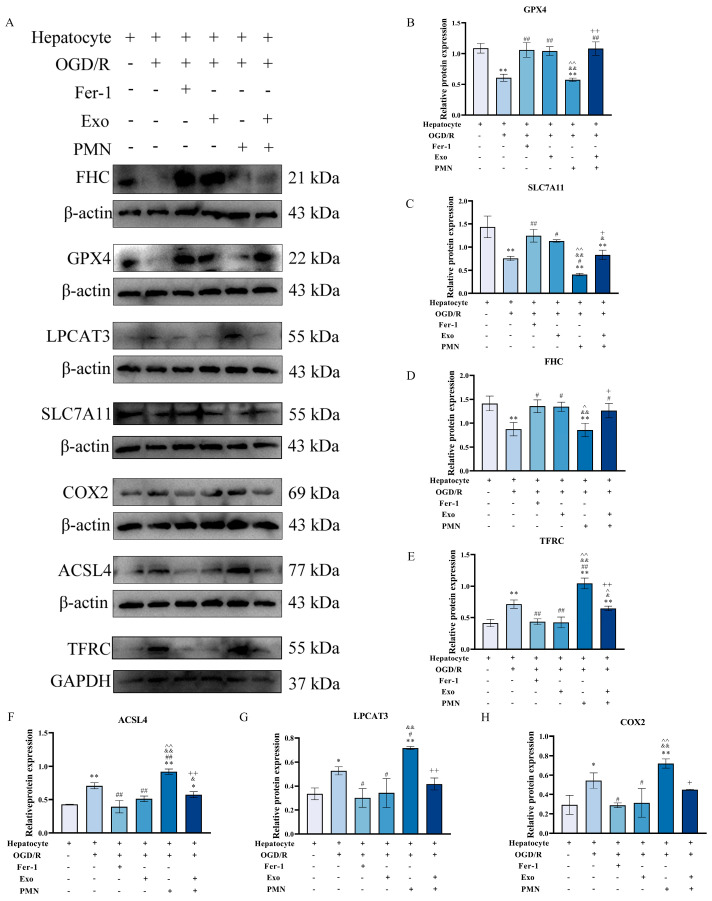
The effects of different interventions on the expression of ferroptosis related proteins in hepatocytes following OGD/R. (**A**) Representative Western blot results of ferroptosis-related proteins in whole-cell lysates from each group of hepatocytes; (**B**) Quantitative analysis of GPX4 protein in whole-cell lysates from each group of hepatocytes; (**C**) Quantitative analysis of SLC7A11 protein in whole-cell lysates from each group of hepatocytes; (**D**) Quantitative analysis of FHC protein in whole-cell lysates from each group of hepatocytes; (**E**) Quantitative analysis of TFRC protein in whole-cell lysates from each group of hepatocytes; (**F**) Quantitative analysis of ACSL4 protein in whole-cell lysates from each group of hepatocytes; (**G**) Quantitative analysis of LPCAT3 protein in whole-cell lysates from each group of hepatocytes; (**H**) Quantitative analysis of COX2 protein in whole-cell lysates from each group of hepatocytes. Data were presented as mean ± SD. * 0.01 < *p* < 0.05, ** *p* < 0.01, versus the Con group. # indicates 0.01 < *p* < 0.05, ## indicates *p* < 0.01, versus the OGD/R group. & 0.01 < *p* < 0.05, && *p* < 0.01, versus the Fer-1 group. ^ 0.01 < *p* < 0.05, ^^ indicates *p* < 0.01, versus the Exo group. + 0.01 < *p* < 0.05, ++ *p* < 0.01, versus the Co group. *n* = 3.

Compared with OGD/R treatment alone, co-culture with PMNs further aggravated hepatocyte injury. Specifically, hepatocyte viability was significantly decreased (Figure 2A; *p* < 0.01), whereas hepatocellular dysfunction was exacerbated, as evidenced by significant increases in ALT, AST, and LDH release levels (Figure 2B–D; *p* < 0.01). Oxidative stress was also markedly enhanced, as reflected by significant decreases in SOD, CAT, and GSH levels (Figure 2E–G; *p* < 0.01) and an increase in MDA content (Figure 2H; 0.01 < *p* < 0.05). In addition, Fe^2+^ fluorescence intensity was significantly increased (Figure 2I,J; *p* < 0.01), and the expression levels of ferroptosis-related proteins were significantly altered (Figure 3B–H; 0.01 < *p* < 0.05). Collectively, these findings indicate that PMN co-culture after reoxygenation aggravated hepatocyte ferroptosis and thereby enhanced hepatocyte injury.

Following ADSCs-Exo intervention, hepatocyte viability was significantly increased compared with that in the co-culture group (Figure 2A; *p* < 0.01). The levels of hepatocellular injury-related indicators, including ALT, AST, and LDH, were significantly decreased (Figure 2B–D; *p* < 0.01). In addition, antioxidant enzyme levels were significantly elevated (Figure 2E,F; *p* < 0.01), whereas MDA content, a marker of lipid peroxidation, was significantly reduced (Figure 2H; *p* < 0.01). Fe^2+^ fluorescence intensity was also markedly attenuated (Figure 2I,J; *p* < 0.01), and the expression levels of ferroptosis-related proteins showed significant changes (Figure 3B–H; 0.01 < *p* < 0.05). Together, these results indicate that ADSCs-Exo alleviated PMN-enhanced hepatocyte ferroptosis after co-culture.

### 3.2. NETs Induce Hepatocyte Ferroptosis

Co-culture of hepatocytes with PMNs after OGD/R further exacerbated hepatocyte ferroptosis. We therefore investigated the mechanism by which PMNs aggravated hepatocyte ferroptosis in this co-culture system.

**Figure 4:**
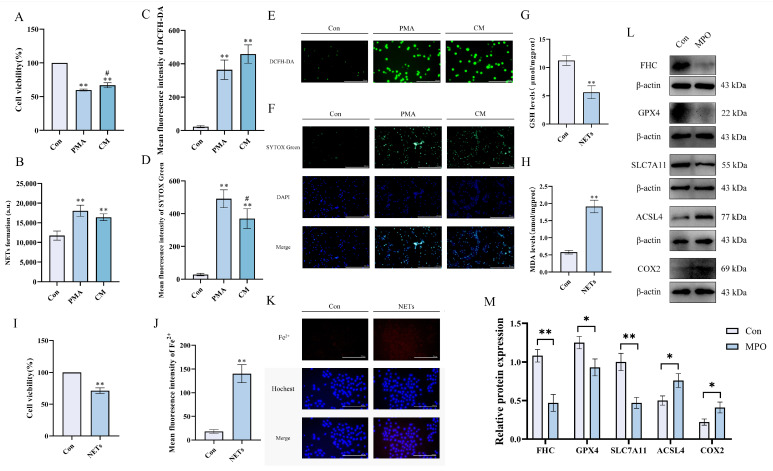
NETs induce hepatocyte ferroptosis. (**A**) Hepatocyte viability assay results in each group; (**B**,**C**) The average fluorescence analysis of DCFH-DA staining in each group of PMNs; (**D**) The average fluorescence analysis of intracellular SYTOX Green staining in each group of PMNs; (**E**) DCFH-DA staining results of intracellular in each group of PMNs (scale bar = 200 μm); (**F**) SYTOX Green staining results of intracellular in each group of PMNs (scale bar = 200 μm); (**G**) GSH assay results in each group of hepatocytes; (**H**) MDA assay results in each group of hepatocytes; (**I**) Hepatocyte viability assay results in each group; (**J**) The average fluorescence analysis of intracellular Fe^2+^ staining in each group of hepatocytes; (**K**) Fe^2+^ staining results of intracellular in each group of hepatocytes (scale bar = 200 μm). (**L**) Representative Western blot results of ferroptosis-related proteins in whole-cell lysates from each group of hepatocytes; (**M**) Quantitative analysis of FHC, SLC7A11, GPX4, ACSL4 and COX2 protein in whole-cell lysates from each group of hepatocytes. Data were presented as mean ± SD. * 0.01 < *p* < 0.05, ** *p* < 0.01, versus the Con group. # 0.01 < *p* < 0.05, versus the PMA group. *n* = 3.

PMNs were cultured in conditioned medium collected from OGD/R-treated hepatocyte (CM group), with 100 nM phorbol 12-myristate 13-acetate (PMA) used as the positive control. Compared with the control group, OGD/R-CM significantly decreased PMN viability (Figure 4A; *p* < 0.01) and increased the mean fluorescence intensity of DCFH-DA staining (Figure 4C,E; *p* < 0.01), indicating enhanced ROS production. In addition, NET formation was assessed by SYTOX Green and propidium iodide (PI) staining using a fluorescence microplate reader. The results showed that the mean fluorescence intensity was markedly increased in the CM group (Figure 4B,D,F; *p* < 0.01). Collectively, these findings indicate that OGD/R-CM promoted ROS production and NET formation in PMNs.

Subsequently, NETs were induced and extracted in vitro and then co-cultured with hepatocytes. Compared with the control group, the NETs group exhibited significantly decreased hepatocyte viability (Figure 4I; *p* < 0.01), reduced GSH levels (Figure 4G; *p* < 0.01), increased MDA levels (Figure 4H; *p* < 0.01), and elevated Fe^2+^ fluorescence intensity (Figure 4J,K; *p* < 0.01). These results demonstrate that NETs induced hepatocyte ferroptosis.

Finally, hepatocytes were treated with MPO, and ferroptosis-related proteins were detected. MPO treatment significantly decreased the protein expression levels of FHC, SLC7A11, and GPX4, while significantly increasing the protein expression levels of ACSL4 and COX2 (Figure 4L,M; 0.01 < *p* < 0.05).

### 3.3. ADSCs-Exo Inhibits NET Formation Induced by OGD/R-CM Through the NADPH/MAPK Pathways

Our data suggested that ADSCs-Exo alleviated PMN-mediated exacerbation of hepatocyte ferroptosis in the co-culture system. Given the potential involvement of NET inhibition, we further investigated the direct effects of ADSCs-Exo on NET formation.

**Figure 5:**
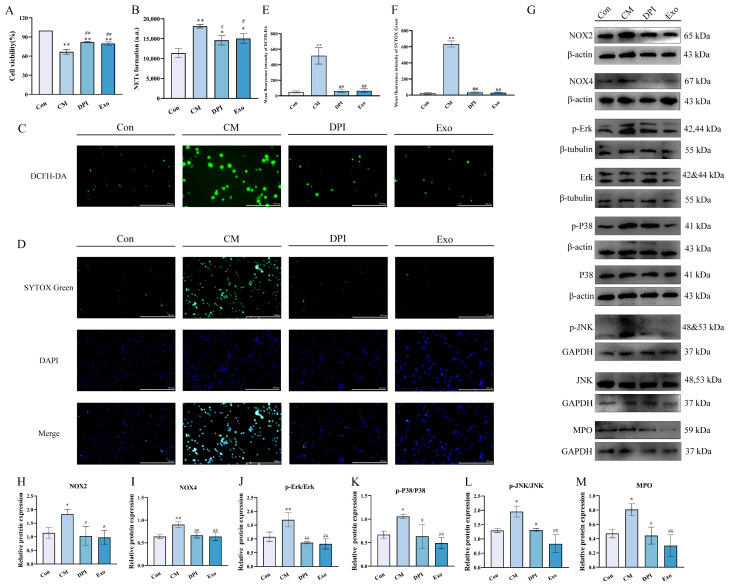
ADSCs-Exo inhibits NET formation induced by OGD/R-CM through the NADPH/MAPK pathways. (**A**) PMN viability assay results in each group; (**B**) NET formation results in each group of PMNs; (**C**) DCFH-DA staining results of intracellular in each group of PMNs (scale bar = 200 μm); (**D**) SYTOX Green staining results of intracellular in each group of PMNs (scale bar = 200 μm); (**E**) The average fluorescence analysis of DCFH-DA staining in each group of PMNs; (**F**) The average fluorescence analysis of intracellular SYTOX Green staining in each group of PMNs; (**G**) Representative Western blot results of NADPH/MAPK pathway-related proteins in whole-cell lysates from each group of PMNs; (**H**) Quantitative analysis of NOX2 protein in whole-cell lysates from each group of PMNs; (**I**) Quantitative analysis of NOX4 protein in whole-cell lysates from each group of PMNs; (**J**) Quantitative analysis of p-Erk/Erk protein in whole-cell lysates from each group of PMNs; (**K**) Quantitative analysis of p-P38/P38 protein in whole-cell lysates from each group of PMNs; (**L**) Quantitative analysis of p-JNK/JNK protein in whole-cell lysates from each group of PMNs; (**M**) Quantitative analysis of myeloperoxidase (MPO) protein in whole-cell lysates from each group of PMNs. Data were presented as mean ± SD. * 0.01 < *p* < 0.05, ** *p* < 0.01, versus the Con group. # 0.01 < *p* < 0.05, ## indicates *p* < 0.01, versus the CM group. *n* = 3.

After preliminary screening, 5 nM diphenyleneiodonium chloride (DPI), an NADPH oxidase inhibitor, was selected as the therapeutic control (Figure S4B) and administered 1 h before conditioned medium (CM) stimulation. Compared with the CM group, treatment with either DPI or ADSCs-Exo significantly restored the CM-induced decrease in PMN viability (Figure 5A; *p* < 0.01). In both the DPI and Exo groups, the number of DCFH-DA-positive cells was reduced (Figure 5C). Consistently, SYTOX Green staining showed a marked reduction in the filamentous and reticular structures of NETs (Figure 5D), accompanied by a significant decrease in NET fluorescence intensity (Figure 5B; *p* < 0.01).

Western blot analysis of proteins in the NADPH/MAPK pathway, which is involved in NET formation, showed that both DPI and ADSCs-Exo significantly downregulated the expression levels of NOX2, NOX4, p-ERK/ERK, p-P38/P38, p-JNK/JNK, and MPO (Figure 5H–M; 0.01 < *p* < 0.05). Collectively, these findings indicate that ADSCs-Exo inhibited CM-induced NET formation, probably through suppression of the NADPH/MAPK signaling pathway.

### 3.4. ADSCs-Exo Attenuates NET Formation After LIRI

In the in vivo experiments, clodronate liposome pretreatment was used to deplete Kupffer cells, serving as a control strategy to reduce Kupffer cell-associated neutrophil recruitment. ADSCs-Exo treatment and the combination of Kupffer cell depletion with ADSCs-Exo administration were further applied to evaluate the therapeutic effects of ADSCs-Exo in miniature pig LIRI.

The efficiency of Kupffer cell depletion is shown in Figure S5. Pretreatment with clodronate liposomes significantly reduced the number of circulating monocytes and hepatic Kupffer cells. In addition, clodronate liposomes showed no obvious hepatotoxicity and had no significant effect on the counts of other blood cells.

Serum inflammatory cytokine analysis showed that, on postoperative day 1, the levels of IL-1β, IL-6, and TNF-α were significantly decreased in the Exo, CI, and CE groups compared with the IRI group (Figure S6A–C; 0.01 < *p* < 0.05). On postoperative day 3, serum IL-1β levels were significantly decreased in the Exo, CI, and CE groups compared with the IRI group (Figure S6A; *p* < 0.01). Serum IL-6 levels were significantly reduced in the Exo and CI groups (Figure S6B; 0.01 < *p* < 0.05), whereas serum TNF-α levels were significantly decreased in the CE group (Figure S6C; 0.01 < *p* < 0.05).

**Figure 6:**
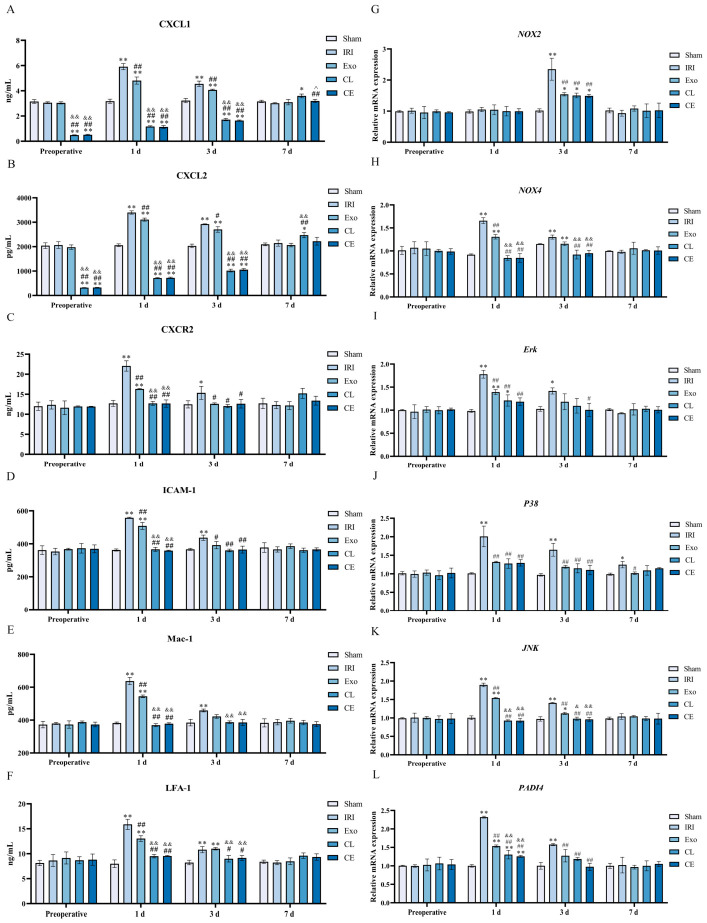
ADSCs-Exo reduced the chemotactic infiltration of PMNs in liver tissue and attenuated the mRNA expression associated with NET formation. (**A**) The CXCL1 levels in the liver; (**B**) The CXCL2 levels in the liver; (**C**) The CXCR2 levels in the liver; (**D**) The ICMA-1 levels in the liver; (**E**) The Mac-1 levels in the liver; (**F**) The LFA-1 levels in the liver; (**G**) The mRNA expression of *NOX2* in the liver; (**H**) The mRNA expression of *NOX4* in the liver; (**I**) The mRNA expression of *Erk* in the liver; (**J**) The mRNA expression of *P38* in the liver; (**K**) The mRNA expression of *JNK* in the liver; (**L**) The mRNA expression of *PADI4* in the liver. Data were presented as mean ± SD. * 0.01 < *p* < 0.05, ** *p* < 0.01, versus the Sham group. # 0.01 < *p* < 0.05, ## *p* < 0.01, versus the IRI group. & 0.01 < *p* < 0.05, && *p* < 0.01, versus the Exo group. ^ 0.01 < *p* < 0.05, versus the CL group. *n* = 3.

Factors associated with PMN chemotaxis, infiltration, and adhesion were then detected in liver tissues. On postoperative day 1, the levels of CXCL1, CXCL2, CXCR2, ICAM-1, Mac-1, and LFA-1 were significantly decreased in the Exo, CI, and CE groups compared with the IRI group (Figure 6A–F; *p* < 0.01). On postoperative day 3, the levels of CXCL1, CXCL2, CXCR2, and ICAM-1 were also significantly reduced in the Exo, CI, and CE groups (Figure 6A–D; 0.01 < *p* < 0.05). Consistently, HE staining of liver tissues (Figure S7A) and serum inflammatory cytokine analysis (Figure S6A–C) showed that ADSCs-Exo, clodronate liposomes, and their combination markedly alleviated LIRI-induced hepatic hemorrhage, necrosis, inflammatory cell infiltration, and congestion after surgery. These findings suggest that ADSCs-Exo reduced chemokine production after LIRI in miniature pigs and alleviated hepatic leukocyte stasis and inflammatory responses associated with neutrophil infiltration.

**Figure 7:**
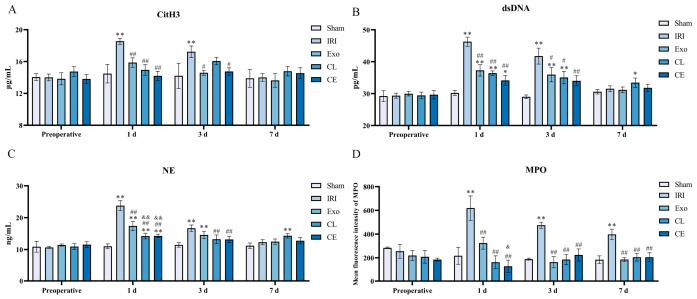
ADSCs-Exo attenuates NET formation after LIRI. (**A**) The CitH3 levels in the liver; (**B**) The dsDNA levels in the liver; (**C**) The NE levels in the liver; (**D**) The average fluorescence analysis of MPO staining in liver; Data are presented as mean ± SD. * 0.01 < *p* < 0.05, ** *p* < 0.01, versus the Sham group. # 0.01 < *p* < 0.05, ## *p* < 0.01, versus the IRI group. & 0.01 < *p* < 0.05, && *p* < 0.01, versus the Exo group. *n* = 3.

**Figure 8:**
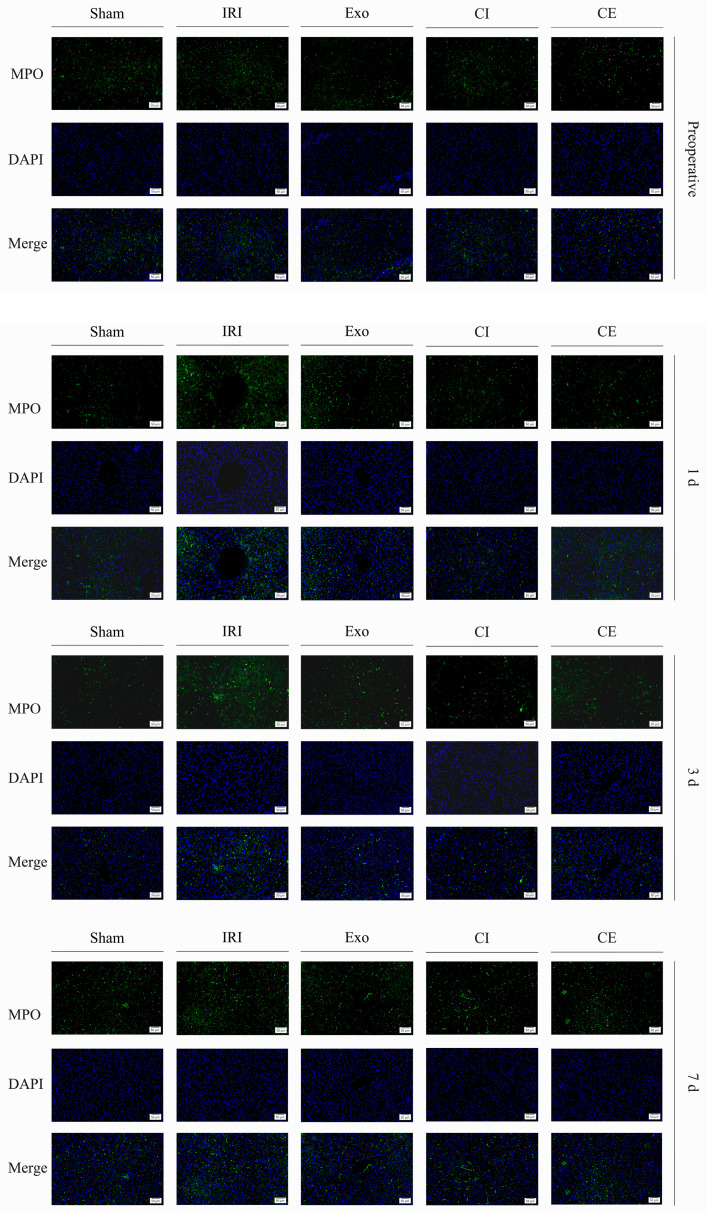
MPO staining results in liver (scale bar = 50 μm).

As shown in Figure 6G–L, the relative expression levels of NET synthesis-related genes in liver tissues, including NOX2, NOX4, Erk, P38, JNK, and PADI4, were significantly lower in the Exo, CI, and CE groups than in the IRI group on postoperative day 1 and/or day 3 (0.01 < *p* < 0.05). Consistently, the levels of NET-related markers, including CitH3, dsDNA, and NE, were significantly reduced in the Exo, CI, and CE groups compared with the IRI group on postoperative day 1 and/or day 3 (Figure 7A–C; 0.01 < *p* < 0.05). Furthermore, MPO immunofluorescence staining and quantitative fluorescence analysis of liver tissues showed a similar decreasing trend (Figure 7D and Figure 8).

### 3.5. ADSCs-Exo Alleviates Ferroptosis After LIRI

**Figure 9:**
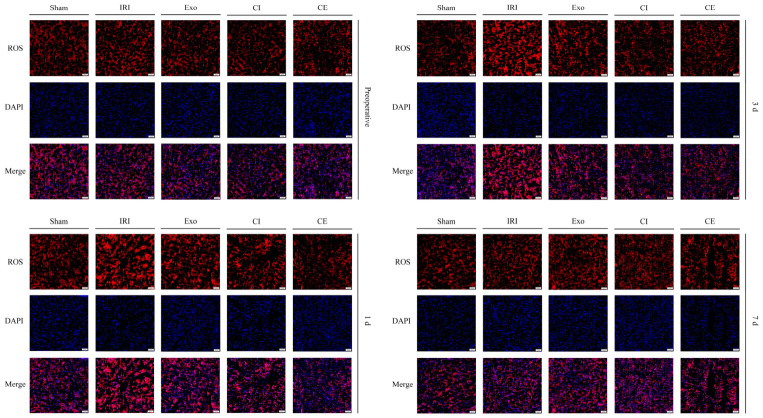
DHE staining results in liver (scale bar = 50 μm).

**Figure 10:**
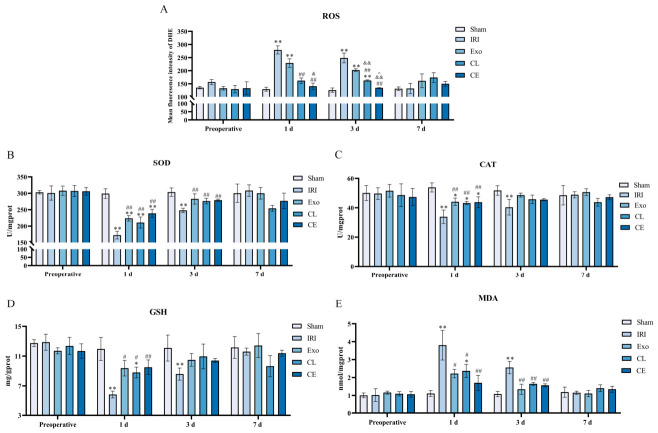
ADSCs-Exo alleviates oxidative stress after LIRI. (**A**) The average fluorescence analysis of DHE staining in liver; (**B**) The SOD levels in the liver; (**C**) The CAT levels in the liver; (**D**) The GSH levels in the liver; (**E**) The MDA levels in the liver Data were presented as mean ± SD. * 0.01 < *p* < 0.05, ** *p* < 0.01, versus the Sham group. # 0.01 < *p* < 0.05, ## *p* < 0.01, versus the IRI group. & 0.01 < *p* < 0.05, && *p* < 0.01, versus the Exo group. ^ 0.01 < *p* < 0.05, versus the CL group. *n* = 3.

DHE staining and quantitative fluorescence analysis of frozen liver sections showed that ROS fluorescence intensity was significantly lower in the CI and CE groups than in the IRI group on postoperative day 1, and was significantly reduced in the Exo, CI, and CE groups on postoperative day 3 (Figure 9 and Figure 10A; *p* < 0.01). Antioxidant enzyme assays showed that SOD and CAT levels or activities were significantly higher in the Exo, CI, and CE groups than in the IRI group on postoperative day 1 (Figure 10B,C; *p* < 0.01). Analysis of lipid peroxidation-related markers further showed that GSH content was significantly increased in the Exo, CI, and CE groups compared with the IRI group on postoperative day 1 (Figure 10D; 0.01 < *p* < 0.05). In contrast, MDA content was significantly decreased in these treatment groups on both postoperative days 1 and 3 (Figure 10E; 0.01 < *p* < 0.05).

**Figure 11:**
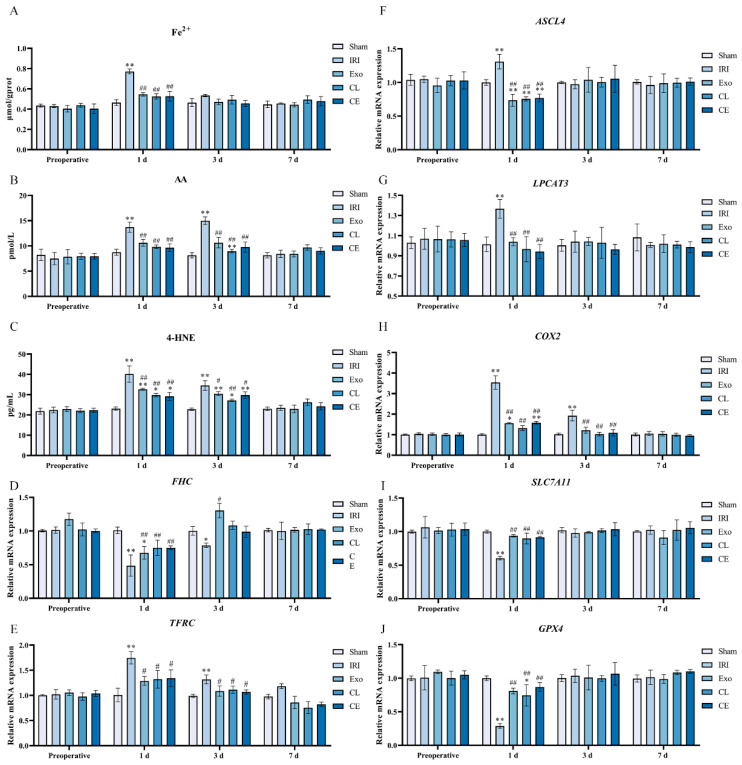
ADSCs-Exo alleviates ferroptosis after LIRI. (**A**) The Fe^2+^ levels in the liver; (**B**) The AA levels in the liver; (**C**) The 4-HNE levels in the liver; (**D**) The mRNA expression of *FHC* in the liver; (**E**) The mRNA expression of *TFRC* in the liver; (**F**) The mRNA expression of *ACSL4* in the liver; (**G**) The mRNA expression of *LPCAT3* in the liver; (**H**) The mRNA expression of *COX2* in the liver; (**I**) The mRNA expression of *SLC7A11* in the liver; (**J**) The mRNA expression of *GPX4* in the liver; Data were presented as mean ± SD. * 0.01 < *p* < 0.05, ** *p* < 0.01, versus the Sham group. # 0.01 < *p* < 0.05, ## *p* < 0.01, versus the IRI group. *n* = 3.

Measurement of Fe^2+^ content in liver tissues showed that, on postoperative day 1, Fe^2+^ levels were significantly lower in the Exo, CI, and CE groups than in the IRI group (Figure 11A; *p* < 0.01). Analysis of lipid peroxidation-related markers further showed that AA and 4-HNE levels were significantly reduced in the Exo, CI, and CE groups compared with the IRI group on postoperative days 1 and 3 (Figure 11B,C; 0.01 < *p* < 0.05).

Detection of ferroptosis-related genes showed that, on postoperative day 1, the expression levels of the iron metabolism-related genes FHC and TFRC were significantly decreased in the Exo, CI, and CE groups compared with the IRI group (Figure 11D,E; 0.01 < *p* < 0.05). The expression levels of the lipid metabolism-related markers ACSL4, COX2, and LPCAT3 were also significantly downregulated (Figure 11F–H; *p* < 0.01), and the expression levels of the GSH metabolism-related genes SLC7A11 and GPX4 were significantly reduced (Figure 11I,J; *p* < 0.01). On postoperative day 3, TFRC and COX2 expression remained significantly lower in the treatment groups than in the IRI group (Figure 11E,H; 0.01 < *p* < 0.05).

Serum liver injury marker analysis showed that ALT and AST levels were significantly lower in the Exo, CI, and CE groups than in the IRI group on postoperative days 1 and 3 (Figure S7B,C; *p* < 0.01). On postoperative day 1, ALT levels were significantly lower in the CI and CE groups than in the Exo group (Figure S7B; 0.01 < *p* < 0.05). Similarly, LDH levels were significantly reduced in the Exo, CI, and CE groups compared with the IRI group (Figure S7D; *p* < 0.01). Moreover, on postoperative day 1, LDH levels were significantly lower in the CI and CE groups than in the Exo group (Figure S7D; *p* < 0.01).

## 4. Discussion

LIRI remains a critical clinical challenge in liver transplantation, hepatectomy, and the management of abdominal trauma [34]. Its pathogenesis is characterized by a complex interplay among oxidative stress, inflammatory cascades, and programmed cell death [35]. Using a miniature pig model that closely resembles human physiology and anatomy, the present study focused on the interaction between NETs and hepatocyte ferroptosis and further elucidated the protective mechanism of ADSCs-Exo against LIRI through modulation of the NADPH/MAPK signaling pathway.

PMNs are key effector cells in the pathogenesis of LIRI, and their infiltration and activation are closely associated with the extent of tissue injury [36]. Our in vitro and in vivo results demonstrated that PMNs exacerbated hepatocyte injury after reperfusion through NET formation. This finding is consistent with previous reports of NET-mediated tissue injury in models of myocardial, renal, and cerebral ischemia–reperfusion injury [13,37,38]. Notably, we demonstrated that NETs induced hepatocyte ferroptosis, as evidenced by disruption of the antioxidant system, including reduced GSH levels and suppressed SOD and CAT activities, elevated intracellular Fe^2+^ levels, and altered expression of key ferroptosis-related proteins. Specifically, NETs downregulated GPX4 and SLC7A11 while upregulating ACSL4 and COX2. Further experimental validation identified MPO, a canonical NET component, as a principal mediator of NET-driven hepatocyte ferroptosis. Although core NET components, such as NE, MPO, and CitH3, have been shown to induce ferroptosis in various pathological conditions, including fatty liver disease [39], Parkinson’s disease [40] and wound healing [41], their role in LIRI has remained largely unexplored. To our knowledge, this study is the first to report this association and directly link NETs to ferroptosis in a large animal model of LIRI, thereby addressing an important gap in the field.

Mesenchymal stem cell-derived exosomes (MSCs-Exo) have emerged as a promising cell-free strategy for tissue repair and have also shown significant efficacy in suppressing NET formation. Human umbilical cord mesenchymal stem cell-derived extracellular vesicles (hUC-MSC-EVs) have been reported to exert nanotherapeutic effects by transferring functional mitochondria to intrahepatic neutrophils, thereby restoring mitochondrial integrity and function in PMNs and inhibiting local NET formation [42]. In addition, MSCs-Exo have been shown to reduce PMN infiltration and NET generation after myocardial ischemia–reperfusion injury [43]. In a mouse model of LPS-induced sepsis-associated acute lung injury, bone marrow mesenchymal stem cell-derived exosomes (BMSCs-Exo) markedly suppressed NET formation by delivering miR-127-5p, which targets CD64 [44]. Furthermore, in a cecal ligation and puncture-induced septic lung injury model, intraperitoneal administration of MSCs-Exo significantly attenuated NET marker elevation, including serum dsDNA and pulmonary CitH3-MPO co-localization, and alleviated histopathological damage in lung tissue [45]. The present study confirmed the therapeutic potential of ADSCs-Exo in alleviating LIRI. In vitro, pretreatment with ADSCs-Exo reversed OGD/R-induced hepatocyte ferroptosis and abolished the exacerbating effect of PMN co-culture on ferroptosis. Mechanistically, we found that ADSCs-Exo inhibited NET formation by suppressing the NADPH/MAPK signaling pathway. Specifically, ADSCs-Exo downregulated key components of the NADPH oxidase complex, including NOX2 and NOX4, and reduced the phosphorylation of core MAPK pathway molecules. These changes led to decreased ROS production and subsequent inhibition of NET release from PMNs. Our findings are consistent with previous studies highlighting the role of NADPH/MAPK signaling in NET formation [46] and extend these insights to a clinically relevant large animal model.

Previous studies have reported that exosomes derived from different MSC sources can alleviate oxidative stress, inflammation, and ferroptosis in LIRI to varying degrees [47,48]. MSCs-Exo have also shown promising potential in inhibiting NET formation [45,49]. However, the suppressive effect of ADSCs-Exo on NETs and the relationship between NETs and hepatocyte ferroptosis in LIRI, particularly in large animal models, have not been fully clarified. In the present study, using a porcine LIRI model, we found that ADSCs-Exo treatment reduced serum levels of inflammatory cytokines, including IL-1β, IL-6, and TNF-α; inhibited the recruitment and infiltration of PMNs, as indicated by decreased levels of CXCL1, CXCL2, and CXCR2 and reduced expression of ICAM-1, Mac-1, and LFA-1; and attenuated NET formation, as reflected by lower levels of CitH3, dsDNA, and NE in liver tissues. Concurrently, ADSCs-Exo alleviated hepatic oxidative stress, as shown by decreased ROS and MDA levels and increased SOD, CAT, and GSH levels, and suppressed ferroptosis, as indicated by reduced Fe^2+^, AA, and 4-HNE levels together with normalized expression of ferroptosis-related genes and proteins. These effects were accompanied by improved liver function, including reduced ALT, AST, and LDH levels, and ameliorated histopathological injury. Notably, the combination of ADSCs-Exo treatment with Kupffer cell depletion did not produce superior therapeutic effects on oxidative stress, inflammation, ferroptosis, or NET-related markers in vivo. This may be attributable to the preferential uptake of exosomes by the reticuloendothelial system [50], with limited uptake by hepatocytes, resulting in comparable therapeutic outcomes among the Exo, CI, and CE groups. These findings further support the concept that LIRI represents an endogenous, localized innate immune-inflammatory response triggered by ischemic-hypoxic stress. Therefore, this study provides new insights into the mitigation of liver injury by targeting neutrophil-mediated detrimental effects after LIRI.

These findings should be interpreted in the context of our previous studies using miniature pig models of minimally invasive hepatectomy combined with LIRI. In earlier work, ADSCs-Exo were shown to ameliorate surgery-related liver injury by attenuating ER stress and UPR activation, including the GRP78, ATF6, IRE1α/XBP1, PERK/eIF2α/ATF4, JNK, and CHOP axes; this established the capacity of ADSCs-Exo to protect hepatocytes from intracellular stress responses in a large-animal setting [51]. A subsequent study demonstrated that ADSCs-Exo also mitigated apoptosis and pyroptosis after hepatic resection combined with LIRI by suppressing TLR4/MyD88/NF-κB/HMGB1-mediated inflammasome activation, the NLRP3-ASC-Caspase-1/GSDMD pyroptotic pathway, and Fas/FasL/Caspase-8 and CytC/APAF1/Caspase-9 apoptotic signaling [52]. Moreover, our recent work further showed that ADSCs-Exo promoted liver regeneration after injury, as reflected by improved liver function and inflammatory balance, increased Ki67, PCNA, Cyclin D1, HGF, STAT3, VEGF, ANG1, and ANG2, and reduced SOCS3 and TGF-β [53]. Therefore, these earlier studies collectively demonstrated that ADSCs-Exo alleviate LIRI through hepatocyte stress regulation, inhibition of inflammatory programmed cell death, and promotion of liver regeneration. Compared with those studies, the present work does not simply repeat the protective effect of ADSCs-Exo in the same disease model. Instead, it extends the previous findings by focusing on a distinct injury-amplifying immune mechanism: the crosstalk between PMNs/NETs and hepatocyte ferroptosis. Specifically, this study identifies MPO as a key NET-derived mediator aggravating hepatocyte ferroptosis and demonstrates that ADSCs-Exo suppress NET formation through inhibition of the NADPH/MAPK pathway, thereby interrupting the NETs-MPO-ferroptosis axis. Thus, the current study represents a mechanistic progression from describing ADSCs-Exo-mediated liver protection/regeneration to defining how ADSCs-Exo modulate neutrophil-driven pathological amplification and ferroptotic hepatocyte injury in a clinically relevant miniature pig model of LIRI.

A major strength of this study is the use of miniature pigs as the experimental model. In liver disease research, mice and rats have several limitations, including substantial differences from humans in pathological manifestations and metabolic mechanisms, as well as an inability to fully replicate clinical conditions [54,55]. Notably, PMNs account for 50–70% of peripheral blood leukocytes in humans but only 10–25% in mice [56], indicating marked interspecies differences in immune response characteristics. In contrast to rodent models, miniature pigs better recapitulate the physiological, anatomical, and immunological features of the human liver [57], thereby improving the clinical translatability of our findings. The laparoscopic partial hepatectomy combined with LIRI model established in this study closely mimics clinical surgical procedures. Moreover, the observed temporal dynamics of inflammatory and ferroptosis-related markers, including peak injury occurring 1–3 days after reperfusion, are consistent with the pathophysiological characteristics of human LIRI. These advantages help overcome major limitations of rodent-based studies and provide a solid preclinical foundation for future clinical studies in liver surgery.

Despite these advances, several limitations should be acknowledged. First, although we confirmed the involvement of the NADPH/MAPK pathway in ADSCs-Exo-mediated suppression of NET formation, the specific bioactive molecules within ADSCs-Exo, such as miRNAs, proteins, or lipids, that target this pathway remain to be elucidated. Second, this study did not evaluate the long-term outcomes of ADSCs-Exo therapy, such as liver regeneration and fibrosis. Future studies should therefore investigate the durability of the therapeutic effects and identify the key functional components responsible for the protective activity of ADSCs-Exo.

In summary, this study provides compelling evidence that ADSCs-Exo alleviate LIRI in a miniature pig model by inhibiting NET formation through suppression of the NADPH/MAPK pathway, thereby attenuating NET-aggravated hepatocyte ferroptosis. These findings deepen our understanding of the pathogenesis of LIRI and support ADSCs-Exo as a potential therapeutic strategy for reducing liver ischemia–reperfusion injury in surgical settings. From a comparative medicine perspective, this study also highlights the value of the porcine model in bridging the translational gap between preclinical rodent studies and human clinical trials. Future research should focus on identifying the active components of ADSCs-Exo and validating their efficacy in larger-scale preclinical studies to facilitate clinical translation.

## 5. Conclusions

This study demonstrated for the first time in a large animal model that ADSCs-Exo attenuated liver ischemia–reperfusion injury by inhibiting NET formation through suppression of the NADPH/MAPK signaling pathway. This effect blocked MPO-mediated aggravation of hepatocyte ferroptosis, thereby reducing hepatic injury. These findings deepen our understanding of the pathogenic mechanisms underlying LIRI and support ADSCs-Exo as a promising cell-free therapeutic strategy for liver injury associated with clinical procedures such as liver transplantation and hepatectomy. Moreover, the use of a miniature pig model enhances the translational relevance of this study and helps bridge the gap between rodent-based preclinical research and human clinical application.

However, although this study confirmed that ADSCs-Exo inhibited NET formation by suppressing the NADPH/MAPK pathway, the key bioactive molecules responsible for this effect remain unidentified. ADSCs-Exo contain various functional biomolecules, including miRNAs, proteins, lipids, and lncRNAs, which may mediate their paracrine effects. Future studies should focus on identifying these specific effector molecules, which may provide novel therapeutic targets for the directed engineering of ADSCs-Exo, improve their therapeutic efficacy, and facilitate their clinical translation.

## Figures and Tables

**Table 1:** Antibodies used for ADSCs and ADSCs-Exo identification.

Name	Manufacturer	Catalog Number	Dilution
CD29	Abcam, Cambridge, UK	ab21845	1:100
CD44	Abcam, Cambridge, UK	ab95138	1:100
CD90	Abcam, Cambridge, UK	ab124527	1:100
CD11b	Biolegend, San Diego, CA, USA	301329	1:100
CD63	Abcam, Cambridge, UK	ab231975	1:1000
CD81	Absin, Shanghai, China	abs126599	1:1000
TSG101	LSBio, Seattle, WA, USA	LS-C408134	1:2000

**Table 2:** Gene-specific primers used for RT-qPCR.

Gene	Forward Primer Sequence (5’ → 3’)	Reverse Primer Sequence (5’ → 3’)
***β-actin***	TCTGGCACCACACCTTCT	TGATCTGGGTCATCTTCTCAC
***GPX4***	TACGGATTCTGGCCTTCCCT	AGTTCCATTTGATAGCATTTCCCAG
***ACSL4***	CCTCTGATTGAAAGCACGAACA	GTGGCATATAAAGTCACAAGAGGA
***COX2***	AATTGCTGGCAGGGTTGCT	GGCGAGGCTTTTCTACCAGA
***FHC***	TACCTGCGCCACAGTCTTC	CGCGCTCCGGTTTCTTGAT
***TFRC***	ACCTCGCTTATTTTGGGCAGA	CTTTGAGCATTTGCCACCTTCT
***LPCAT3***	CTATGGGGCCTTCTTGGTGG	GCAGGTATGGTGCTGTTTGG
***SLC7A11***	CTGGCATTTGGACGCTACA	CAACAGTTCCTCGGCACTAA
***NOX2***	CCACGATTCACACCATTGCC	ACGTACAATTCGCTCGGCT
***NOX4***	GCTAGGACGCCAAAGGCTATT	TTAGGCACAATCCCCCAGCA
***Erk***	GCTGAACCACATTCTGGGTATTC	GCTCAAAGGAGTCAAGGTGGA
***p38***	CCCCAGCTCAAGGCAGTTTCTA	CCTCCTGGCTTCAGAATGGTGG
***JNK***	GGGCAGCGTCTCTGTTACTCA	CTGCTACCTGGAGATTATACTGGC
***PADI4***	GTGGTCTTCGACTCACCGAG	CAGTCACCCTCCCTGTTTGG

**Table 3:** Antibodies used for Western blotting analysis.

Name	Manufacturer	Catalog Number	Dilution	Reactivity
**β-actin**	Proteintech, Wuhan, China	81115-1-RR	1:10000	Rabbit
**β-tubulin**	HUABIO, Hangzhou, China	ST1602-4	1:20000	Rabbit
**GAPDH**	Proteintech	60004-1-Ig	1:50000	Mouse
**GPX4**	HUABIO	ET1706-45	1:10000	Rabbit
**COX2**	Abmart, Shanghai, China	TA7003	1:1000	Rabbit
**FHC**	Abclonal, Wuhan, China	A19544	1:1000	Rabbit
**TFRC**	HUABIO	ET1702-06	1:1000	Rabbit
**ACSL4**	Proteintech	2240-1-AP	1:1000	Rabbit
**SLC7A11**	BYabscience, Nanjing, China	Byab-17924	1:1000	Rabbit
**LPCAT3**	Abmart	OK53132	1:1000	Rabbit
**NOX2**	HUABIO	ET1611-44	1:2000	Rabbit
**NOX4**	HUABIO	ET1607-4	1:2000	Rabbit
**Erk**	Proteintech	11257-1-AP	1:2000	Rabbit
**p-Erk**	Proteintech	28733-1-AO	1:1000	Rabbit
**P38**	Proteintech	66234-1-AP	1:2000	Rabbit
**p-P38**	Proteintech	28796-1-AP	1:1000	Rabbit
**JNK**	CST	9252T	1:1000	Rabbit
**p-JNK**	BYabscience	BYab-17797	1:1000	Rabbit
**MPO**	Proteintech	22225-1-AP	1:1000	Rabbit

## Data Availability

The original contributions presented in this study are included in the article/Supplementary Materials. Further inquiries can be directed to the corresponding author.

## Supplementary Materials

The following supporting information can be downloaded at: https://www.mdpi.com/article/10.3390/cells15111040/s1, Figure S1. Identification of ADSCs, ADSCs-Exo, hepatocytes, and PMNs. Figure S2. Flow cytometry analysis of ADSCs. Figure S3. Hepatocytes effectively uptake ADSCs- Exo (scale bar = 20 μm). Figure S4. Results of the optimal effective concentrations and time points of Fer-1 and DPI. Figure S5. Clodronate liposomes significantly reduced the number of monocytes in the blood and Kupffer cells in the liver. Figure S6. ADSCs-Exo alleviates the inflammatory response following LIRI. Figure S7. ADSCs-Exo alleviates liver injury following LIRI.

## Abbreviations

LIRI: Liver ischemia-reperfusion injury; PMNs: polymorphonuclear neutrophils; NETs: neutrophil extracellular traps; ROS: reactive oxygen species; Fer-1: ferrostatin-1; ADSCs-Exo: Adipose-derived mesenchymal stem cell-derived exosomes; ADSCs: adipose-derived mesenchymal stem cells; OGD/R: oxygen-glucose deprivation/reoxygenation; PBS: phosphate-buffered saline; LSCM: laser scanning confocal microscope; D-PBS: Dulbecco’s phosphate buffered saline; CCK-8: cell counting kit-8; SD: standard deviation; ANOVA: analyzed by one-way analysis of variance; LSD: Least Significant Difference; PMA: Phorbol 12-myristate 13-acetate; PI: propidium iodide; DPI: diphenyleneiodonium; MSCs-Exo: Mesenchymal stem cell-derived exosomes; hUC-MSC-EVs: Human umbilical cord mesenchymal stem cell-derived exomes; BMSCs-Exo: bone marrow mesenchymal stem cell-derived exosomes.

## References

[1] Konishi T., Lentsch A.B. (2017). Hepatic Ischemia/Reperfusion: Mechanisms of Tissue Injury, Repair, and Regeneration. Gene Expr..

[2] Li Q., Qin X., Wang L., Hu D., Liao R., Yu H., Wu Z., Liu Y. (2025). Multi-time point transcriptomics and metabolomics reveal key transcription and metabolic features of hepatic ischemia-reperfusion injury in mice. Genes Dis..

[3] Galaris D., Barbouti A., Korantzopoulos P. (2006). Oxidative stress in hepatic ischemia-reperfusion injury: The role of antioxidants and iron chelating compounds. Curr. Pharm. Des..

[4] Abu-Amara M., Yang S.Y., Tapuria N., Fuller B., Davidson B., Seifalian A. (2010). Liver ischemia/reperfusion injury: Processes in inflammatory networks—A review. Liver Transplant..

[5] Hirao H., Nakamura K., Kupiec-Weglinski J.W. (2022). Liver ischaemia-reperfusion injury: A new understanding of the role of innate immunity. Nat. Rev. Gastroenterol. Hepatol..

[6] Jaeschke H., Farhood A., Smith C.W. (1990). Neutrophils contribute to ischemia/reperfusion injury in rat liver in vivo. FASEB J..

[7] Jorch S.K., Kubes P. (2017). An emerging role for neutrophil extracellular traps in noninfectious disease. Nat. Med..

[8] Cristinziano L., Modestino L., Antonelli A., Marone G., Simon H.U., Varricchi G., Galdiero M.R. (2022). Neutrophil extracellular traps in cancer. Semin. Cancer Biol..

[9] Burgener S.S., Schroder K. (2020). Neutrophil Extracellular Traps in Host Defense. Cold Spring Harb. Perspect. Biol..

[10] Wilson A.S., Randall K.L., Pettitt J.A., Ellyard J.I., Blumenthal A., Enders A., Quah B.J., Bopp T., Parish C.R., Brüstle A. (2022). Neutrophil extracellular traps and their histones promote Th17 cell differentiation directly via TLR2. Nat. Commun..

[11] de Bont C.M., Boelens W.C., Pruijn G.J.M. (2019). NETosis, complement, and coagulation: A triangular relationship. Cell. Mol. Immunol..

[12] Zhang Z., Wang Y., Li T., Wang H. (2025). NETosis in myocardial ischemia-reperfusion injury: From mechanisms to therapies (Review). Biomed. Rep..

[13] Wu X., You D., Cui J., Yang L., Lin L., Chen Y., Xu C., Lian G., Wan J. (2022). Reduced Neutrophil Extracellular Trap Formation During Ischemia Reperfusion Injury in C3 KO Mice: C3 Requirement for NETs Release. Front. Immunol..

[14] Luo H., Guo H., Zhou Y., Fang R., Zhang W., Mei Z. (2023). Neutrophil Extracellular Traps in Cerebral Ischemia/Reperfusion Injury: Friend and Foe. Curr. Neuropharmacol..

[15] Zhu C., Shi S., Jiang P., Huang X., Zhao J., Jin Y., Shen Y., Zhou X., Liu H., Cai J. (2023). Curcumin Alleviates Hepatic Ischemia-Reperfusion Injury by Inhibiting Neutrophil Extracellular Traps Formation. J. Investig. Surg..

[16] Dixon S.J., Lemberg K.M., Lamprecht M.R., Skouta R., Zaitsev E.M., Gleason C.E., Patel D.N., Bauer A.J., Cantley A.M., Yang W.S. (2012). Ferroptosis: An iron-dependent form of nonapoptotic cell death. Cell.

[17] Golfeyz S., Lewis S., Weisberg I.S. (2018). Hemochromatosis: Pathophysiology, evaluation, and management of hepatic iron overload with a focus on MRI. Expert Rev. Gastroenterol. Hepatol..

[18] Hara Y., Yanatori I., Tanaka A., Kishi F., Lemasters J.J., Nishina S., Sasaki K., Hino K. (2020). Iron loss triggers mitophagy through induction of mitochondrial ferritin. EMBO Rep..

[19] Zhou L., Han S., Guo J., Qiu T., Zhou J., Shen L. (2022). Ferroptosis-A New Dawn in the Treatment of Organ Ischemia-Reperfusion Injury. Cells.

[20] Si J., Wang J., Dai H., Lv T., Zhao S., Chen W., Li L., Ding S., He Y. (2025). Mechanistic insights into adipose-derived stem cells and exosomes in ischemia-reperfusion injury repair: From shared pathways to organ-specific therapeutics. Front. Cell Dev. Biol..

[21] Gong Y., Dai H., Liu W., Liao R., Chen H., Zhang L., Wang X., Chen Z. (2023). Exosomes derived from human adipose-derived stem cells alleviate hepatic ischemia-reperfusion (I/R) injury through the miR-183/ALOX5 axis. FASEB J..

[22] Zhang Q., Piao C., Xu J., Wang Y., Liu T., Ma H., Wang H. (2023). ADSCs-exo attenuates hepatic ischemia-reperfusion injury after hepatectomy by inhibiting endoplasmic reticulum stress and inflammation. J. Cell. Physiol..

[23] Ma Y., Liu T., Li P., Cao L., Lu X., Wang H. (2025). Exosomes derived from ALR-modified adipose mesenchymal stem cells mediate hepatoprotective effects on hepatic ischemia-reperfusion injury by promoting regeneration and protecting mitochondria. Stem Cell Res. Ther..

[24] Peter A.K., Crocini C., Leinwand L.A. (2017). Expanding our scientific horizons: Utilization of unique model organisms in biological research. EMBO J..

[25] Abu-Toamih Atamni H.J., Iraqi F.A. (2019). Efficient protocols and methods for high-throughput utilization of the Collaborative Cross mouse model for dissecting the genetic basis of complex traits. Anim. Models Exp. Med..

[26] Dorandeu F., Mikler J.R., Thiermann H., Tenn C., Davidson C., Sawyer T.W., Lallement G., Worek F. (2007). Swine models in the design of more effective medical countermeasures against organophosphorus poisoning. Toxicology.

[27] Arora D., Park J.E., Lim D., Cho I.C., Kang K.S., Kim T.H., Park W. (2022). Multi-omics approaches for comprehensive analysis and understanding of the immune response in the miniature pig breed. PLoS ONE.

[28] Lu X., Wang Y., Piao C., Li P., Cao L., Liu T., Ma Y., Wang H. (2024). Exosomes Derived from Adipose Mesenhymal Stem Cells Ameliorate Lipid Metabolism Disturbances Following Liver Ischemia-Reperfusion Injury in Miniature Swine. Int. J. Mol. Sci..

[29] Risha Y., Minic Z., Ghobadloo S.M., Berezovski M.V. (2020). The proteomic analysis of breast cell line exosomes reveals disease patterns and potential biomarkers. Sci. Rep..

[30] Altıntaş Ö., Saylan Y. (2023). Exploring the Versatility of Exosomes: A Review on Isolation, Characterization, Detection Methods, and Diverse Applications. Anal. Chem..

[31] Dave S., Mathur M., Bhatnagar V. (2000). Hepatocyte isolation and transplantation in syngenic rats. Trop. Gastroenterol. Off. J. Dig. Dis. Found..

[32] Zhang Q., Ge Y., Li H., Bai G., Jiao Z., Kong X., Meng W., Wang H. (2018). Effect of hydrogen-rich saline on apoptosis induced by hepatic ischemia reperfusion upon laparoscopic hepatectomy in miniature pigs. Res. Vet. Sci..

[33] Hashimoto S., Honda M., Takeichi T., Sakisaka M., Narita Y., Yoshii D., Uto K., Sakamoto S., Inomata Y. (2018). Intravital imaging of neutrophil recruitment in intestinal ischemia-reperfusion injury. Biochem. Biophys. Res. Commun..

[34] Sakai N., Van Sweringen H.L., Schuster R., Blanchard J., Burns J.M., Tevar A.D., Edwards M.J., Lentsch A.B. (2012). Receptor activator of nuclear factor-κB ligand (RANKL) protects against hepatic ischemia/reperfusion injury in mice. Hepatology.

[35] de Oliveira T.H.C., Gonçalves G.K.N. (2025). Liver ischemia reperfusion injury: Mechanisms, cellular pathways, and therapeutic approaches. Int. Immunopharmacol..

[36] Chen Y.X., Sato M., Kawachi K., Abe Y. (2006). Neutrophil-mediated liver injury during hepatic ischemia-reperfusion in rats. Hepatobiliary Pancreat. Dis. Int. HBPD INT.

[37] Xia H., Yan J.C. (2021). Research progress on the association between neutrophil extracellular traps and myocardial ischemia reperfusion. Zhonghua Xin Xue Guan Bing Za Zhi.

[38] Peng J., Huang Y., He T., Zhan Y., Liu J. (2025). NLRX1 mediated impaired microglial phagocytosis of NETs in cerebral ischemia and reperfusion injury. Cell Death Differ..

[39] Yang Q., Shen X., Luo Y., Li R., Meng X., Xu P., Liu X., Bian D., Wang J., Shi J. (2025). ELANE enhances KEAP1 protein stability and reduces NRF2-mediated ferroptosis inhibition in metabolic dysfunction-associated fatty liver disease. Cell Death Dis..

[40] Boonpraman N., Yoon S., Kim C.Y., Moon J.S., Yi S.S. (2023). NOX4 as a critical effector mediating neuroinflammatory cytokines, myeloperoxidase and osteopontin, specifically in astrocytes in the hippocampus in Parkinson’s disease. Redox Biol..

[41] Zhao H., Liu Y. (2025). Neutrophil extracellular traps induce fibroblast ferroptosis via IRE1α/XBP1-mediated ER stress to impair diabetic wound healing. Free Radic. Biol. Med..

[42] Lu T., Zhang J., Cai J., Xiao J., Sui X., Yuan X., Li R., Li Y., Yao J., Lv G. (2022). Extracellular vesicles derived from mesenchymal stromal cells as nanotherapeutics for liver ischaemia-reperfusion injury by transferring mitochondria to modulate the formation of neutrophil extracellular traps. Biomaterials.

[43] Feng Y., Bao X., Zhao J., Kang L., Sun X., Xu B. (2024). MSC-Derived Exosomes Mitigate Myocardial Ischemia/Reperfusion Injury by Reducing Neutrophil Infiltration and the Formation of Neutrophil Extracellular Traps. Int. J. Nanomed..

[44] Zheng X.L., Gu W.J., Zhang F., Zhao F.Z., Li L.Z., Huang H.Y., Li L.J., Yi Y.H., Yin H.Y., Xu J. (2023). Exosomal miR-127-5p from BMSCs alleviated sepsis-related acute lung injury by inhibiting neutrophil extracellular trap formation. Int. Immunopharmacol..

[45] Zou T., Lu J., Zhu Y., Xu Y., Sun Y. (2025). Mesenchymal stem cell-derived exosomes improved septic lung injury by reducing excessive NETs formation and alleviating inflammatory response. Allergol. Immunopathol..

[46] Arumugam S., Girish Subbiah K., Kemparaju K., Thirunavukkarasu C. (2018). Neutrophil extracellular traps in acrolein promoted hepatic ischemia reperfusion injury: Therapeutic potential of NOX2 and p38MAPK inhibitors. J. Cell. Physiol..

[47] Gong Y., You Q., Yuan X., Zeng F., Zhang F., Xiao J., Chen H., Liu Y., Wang T., Yan X. (2025). Mesenchymal stem cell-derived extracellular vesicles attenuate ferroptosis in aged hepatic ischemia/reperfusion injury by transferring miR-1275. Redox Biol..

[48] Miao L., Yu C., Guan G., Luan X., Jin X., Pan M., Yang Y., Yan J., Chen P., Di G. (2024). Extracellular vesicles containing GAS6 protect the liver from ischemia-reperfusion injury by enhancing macrophage efferocytosis via MerTK-ERK-COX2 signaling. Cell Death Discov..

[49] Chen L., Liu Y., Wang Z., Zhang L., Xu Y., Li Y., Zhang L., Wang G., Yang S., Xue G. (2023). Mesenchymal stem cell-derived extracellular vesicles protect against abdominal aortic aneurysm formation by inhibiting NET-induced ferroptosis. Exp. Mol. Med..

[50] Kusuma R.J., Manca S., Friemel T., Sukreet S., Nguyen C., Zempleni J. (2016). Human vascular endothelial cells transport foreign exosomes from cow’s milk by endocytosis. Am. J. Physiol. Cell Physiol..

[51] Wang Y., Liu T., Jiao G., Lv Y., Piao C., Lu X., Ma H., Wang H. (2023). Exosomes from adipose-derived mesenchymal stem cells can attenuate liver injury caused by minimally invasive hemihepatectomy combined with ischemia-reperfusion in minipigs by modulating the endoplasmic reticulum stress response. Life Sci..

[52] Wang Y., Piao C., Liu T., Lu X., Ma Y., Zhang J., Liu G., Wang H. (2023). Effects of the exosomes of adipose-derived mesenchymal stem cells on apoptosis and pyroptosis of injured liver in miniature pigs. Biomed. Pharmacother..

[53] Wang Y., Piao C., Liu T., Lu X., Ma Y., Zhang J., Ma H., Wang H. (2024). Exosomes Derived from Adipose Mesenchymal Stem Cells Promote Regeneration of Injured Liver in Minipigs. Int. J. Mol. Sci..

[54] Liu S.X., Du Y.C., Zeng T. (2021). A mini-review of the rodent models for alcoholic liver disease: Shortcomings, application, and future prospects. Toxicol. Res..

[55] Xu Z., Kang Q., Yu Z., Tian L., Zhang J., Wang T. (2021). Research on the Species Difference of the Hepatotoxicity of Medicine Based on Transcriptome. Front. Pharmacol..

[56] Hidalgo A., Chilvers E.R., Summers C., Koenderman L. (2019). The Neutrophil Life Cycle. Trends Immunol..

[57] Swindle M.M., Makin A., Herron A.J., Clubb F.J., Frazier K.S. (2012). Swine as models in biomedical research and toxicology testing. Vet. Pathol..

