# Supplementary material for: ADSCs-Exo Attenuate NET Formation via the NADPH/MAPK Pathway and Mitigate NETs-Mediated Exacerbation of Hepatocyte Ferroptosis in a Miniature Pig Model of LIRI
Source: Cells. 2026 Jun 5;15(11):1040. doi: 10.3390/cells15111040 (PMC13257348; doi:10.3390/cells15111040)
Supplement: Supplementary file 1 [file cells-15-01040-s001.zip › cells-4320975-supplementary.pdf]

Supplementary Materials

Identification of ADSCs, ADSCs-Exo, hepatocytes, and PMNs

Under microscopic examination, ADSCs exhibited a spindle-shaped morphology and adhered to the culture surface with a whirlpool-like growth pattern (Figure S1 A). Flow cytometric analysis revealed high expression of the positive surface markers CD29, CD44, and CD90, along with low expression of the immune cell marker CD11b (Figure S1). When cultured in specific induction media, ADSCs differentiated into osteoblasts (Figure S1 B) and adipocytes (Figure S1 C), confirming their multipotent differentiation capacity.

ADSCs-Exo isolated by ultracentrifugation displayed a cup-shaped or biconcave discoid morphology with a characteristic bilayer membrane structure under transmission electron microscopy (Figure S1 J). Western blot analysis confirmed the consistent expression of exosome-specific markers CD63, CD81, and TSG101 (Figure S1 K). In addition, NTA indicated that the majority of ADSCs-Exo particles ranged in size from 30 to 150 nm (Figure S1 L).

Under light microscopy, hepatocytes adhered to the culture surface and exhibited a polygonal morphology with tight intercellular junctions and an island-like arrangement (Figure S1 D). Periodic acid–Schiff (PAS) staining showed pink cytoplasmic staining and blue nuclei (Figure S1 E). Immunofluorescence staining confirmed positive expression of CK-18 (Figure S1 G–I). In Wright–Giemsa-stained neutrophil smears, light microscopy revealed cells with multi-lobulated nuclei (Figure S1 F).

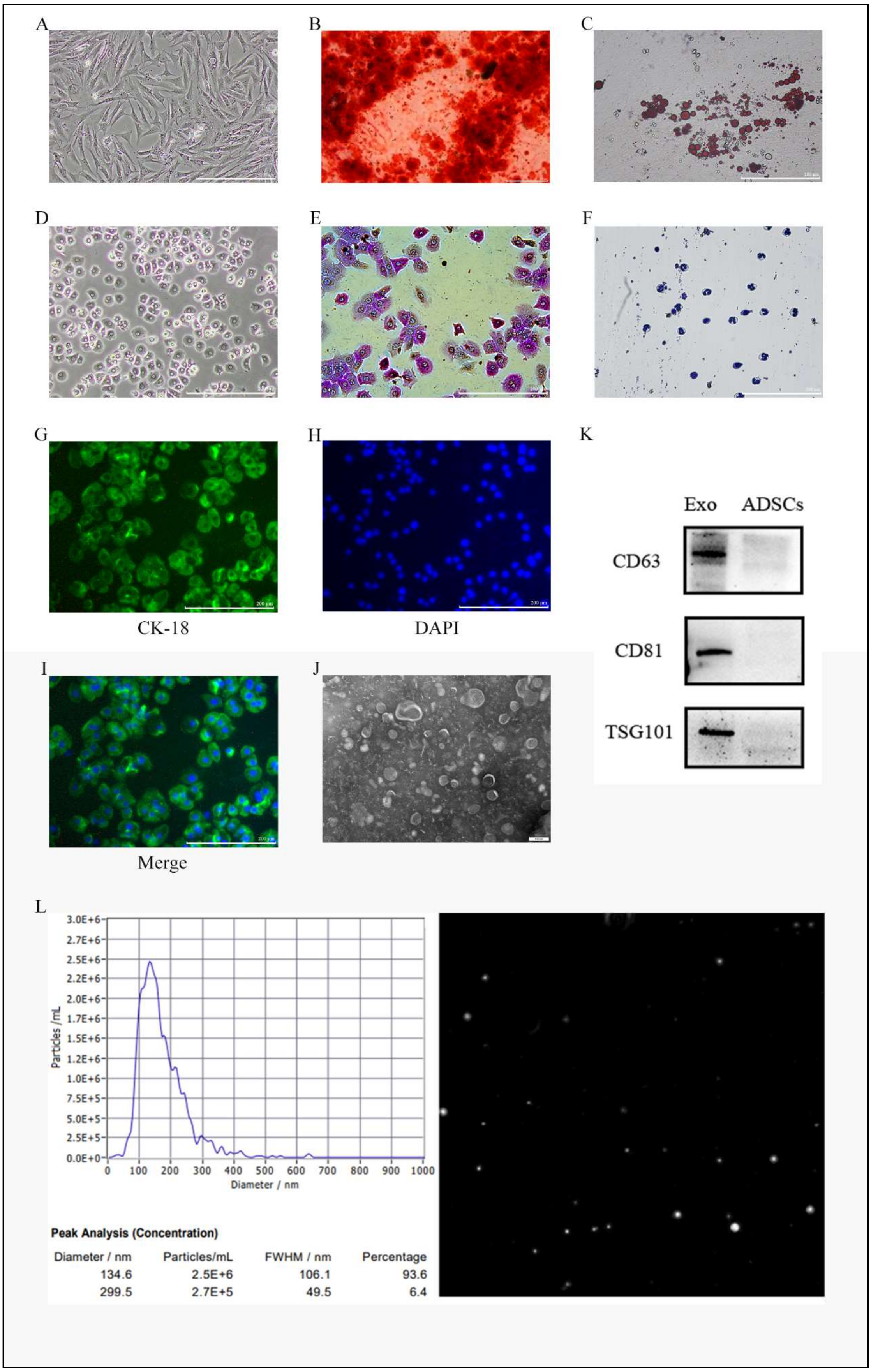

Figure S1. Identification of ADSCs, ADSCs-Exo, hepatocytes, and PMNs.

A: The morphology of ADSCs was observed by light microscopy (scale bar = 200 μm); B: ; Alizarin Red S-positive calcium nodules (scale bar = 200 μm); C: Oil-red O stained droplets (scale bar = 200 μm); D: The morphology of hepatocytes was observed by light microscopy scale bar = 200 μm); E: PAS stained glycogen granules (scale bar = 200 μm); F: The Wright-Giemsa stained of PMNs (scale bar = 200 μm); G-I: The CK-18 immunofluorescence stained of hepatocytes

J: Morphology of ADSCs-Exo by TEM (scale bar = 100 nm); K: Western blot of CD63, CD81 and TSG101 protein expression in ADSCs-Exo; L: NTA of ADSCs
-Exo.

Flow cytometry results demonstrated that ADSCs exhibited low expression of the hematopoietic cell marker CD11b, with a positive rate of 0.37%. In contrast, they highly expressed mesenchymal stem cell (MSC)-specific markers, including CD29, CD44, and CD90, at positive rates of 97.5%, 95.1%, and 99.9%, respectively.

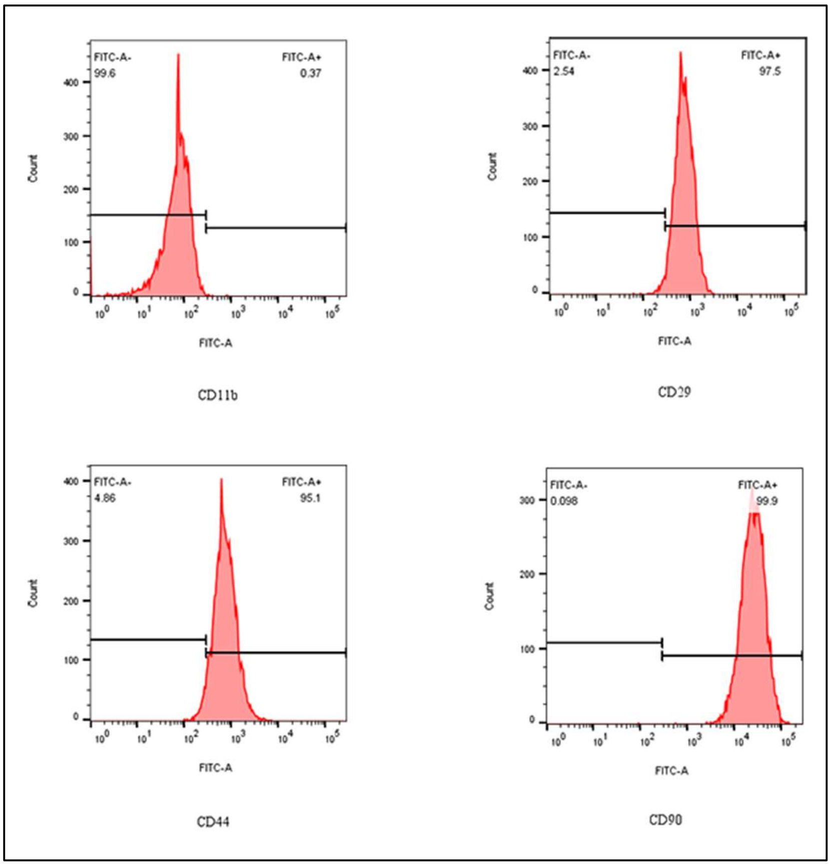

Figure S2. Flow cytometry analysis of ADSCs.

Hepatocytes effectively uptake ADSCs- Exo

The results demonstrated that ADSCs-Exo can be stably labeled with Aco600, and

the labeled ADSCs-Exo were efficiently internalized by hepatocytes following co-incubation.

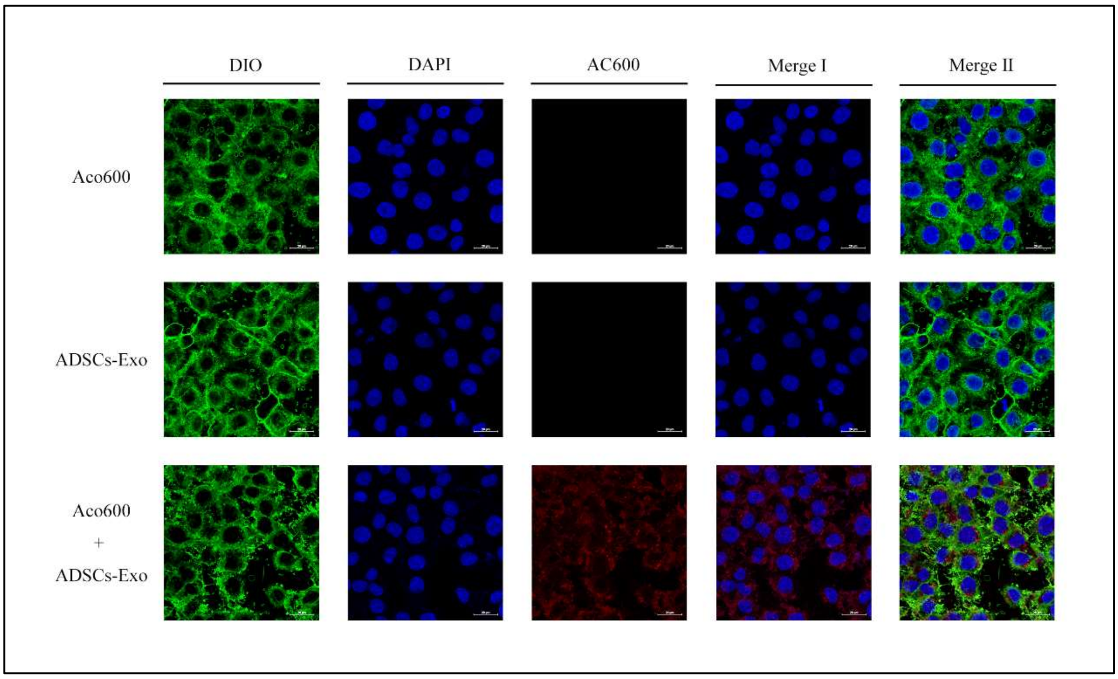

Figure S3. Hepatocytes effectively uptake ADSCs- Exo (scale bar = 20 μm).

Results of the optimal effective concentrations and time points of Fer-1 and DPI

CCK-8 assay results showed that the average viability of hepatocytes was the highest (87.03%) when pretreated with 60 nM Fer-1 at 6 hours prior to oxygen-glucose deprivation/reperfusion (OGD/R) treatment. Similarly, the average viability of neutrophils reached the maximum (93.52%) following pretreatment with 5 nM diphenyleneiodonium (DPI) at 1 hour before cultivation with OGD/R-conditioned medium (OGD/R-CM).

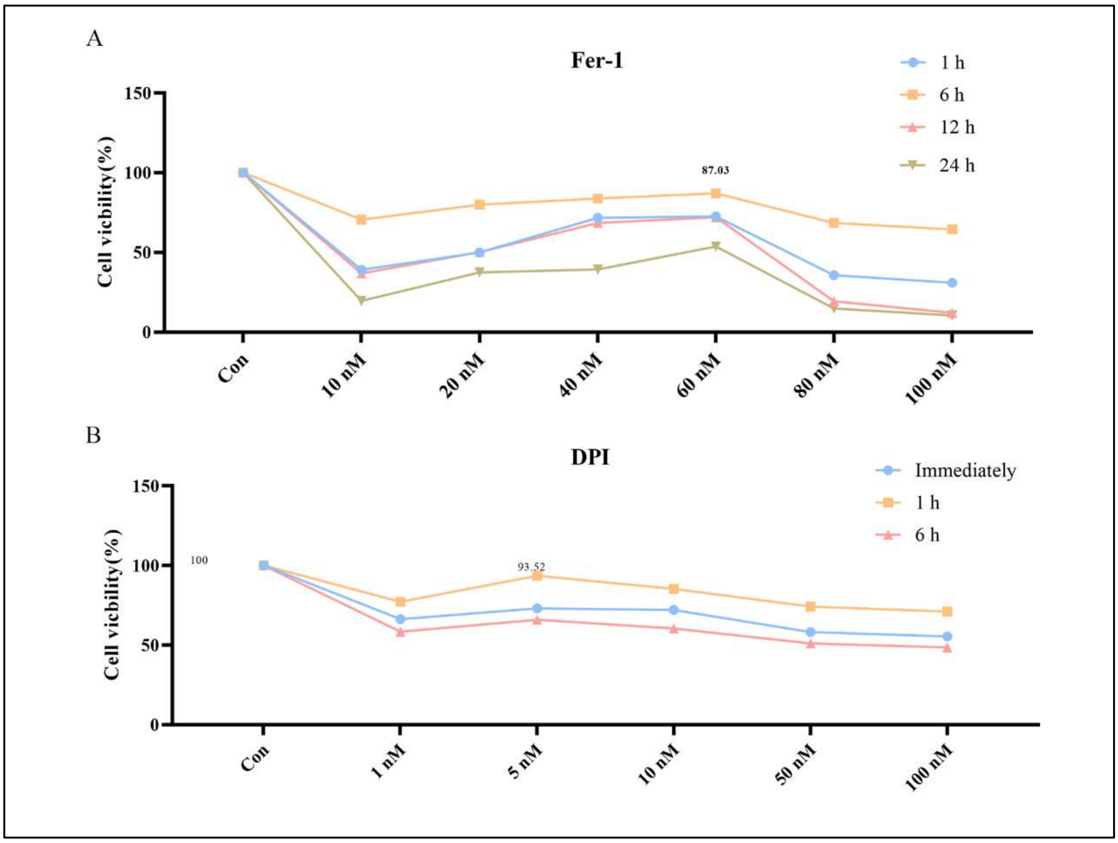

Figure S4. Results of the optimal effective concentrations and time points of Fer-1 and DPI.

Clodronate disodium liposome pretreatment effectively depletes Kupffer cells in liver tissues

Pretreatment with clodronate liposomes significantly reduced the number of MO in the blood (Figure S5A, $p < 0.01$), but had no obvious effect on the counts of other blood cells, NE, LY and RBC (Figures S5B–D). The results of serum liver function tests showed that pretreatment exerted no significant influence on the levels of alanine ALT, AST, LDH, ALP and TBIL (Figures S5E–I, $p > 0.05$). Detection of the CD68 gene in liver tissue revealed that the relative expression level of the CD68 gene in the CI and CE groups was significantly lower than that in the Sham, IRI and Exo groups before LIRI surgery, as well as on days 1 and 3 postoperatively (Figure S5J, $p < 0.01$).

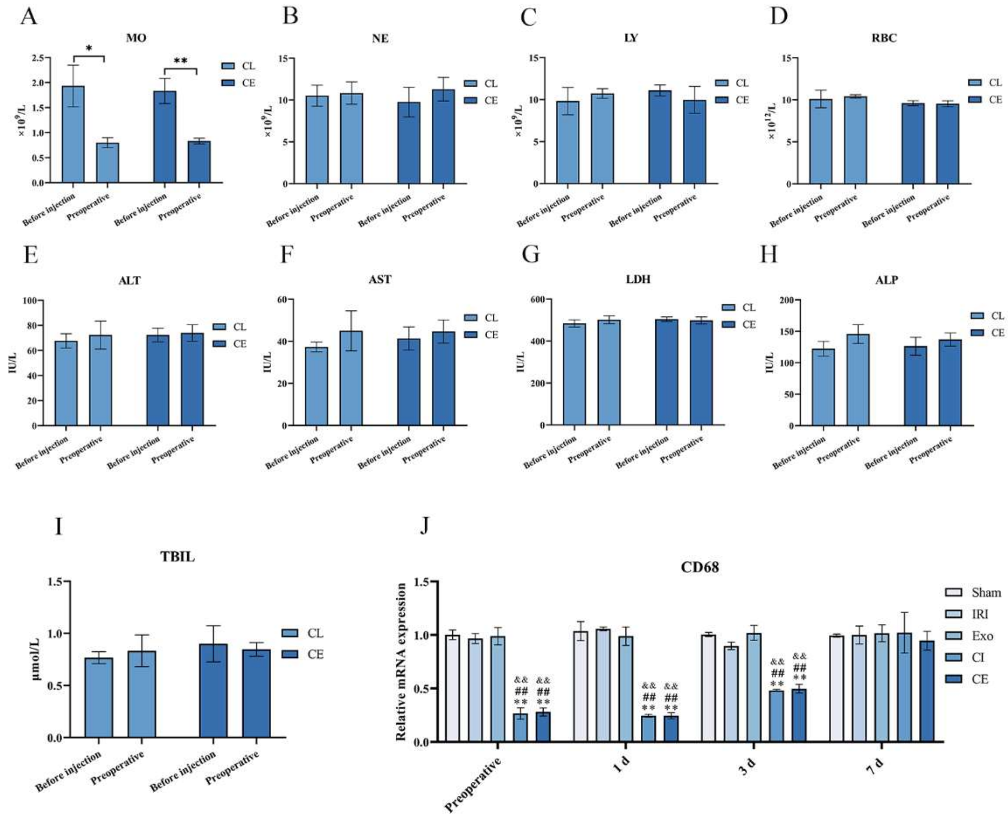

Figure S5. Clodronate liposomes significantly reduced the number of monocytes in the blood and Kupffer cells in the liver.

A: Number of MO in the blood of the CI and CE groups before and after treatment; B: Number of NE in the blood of the CI and CE groups before and after treatment; C: Number of LY in the blood of the CI and CE groups before and after treatment; D: Number of RBC in the blood of the CI and CE groups before and after treatment; E: Serum ALT level in the CI and CE groups before and after treatment; F: Serum AST level in the CI and CE groups before and after treatment; G: Serum LDH level in the CI and CE groups before and after treatment; H: Serum ALP level in the CI and CE groups before and after treatment; I: Serum TBIL level in the CI and CE groups before and after treatment; J: CD68 mRNA expression in liver tissues of the CI and CE groups: Preoperatively, and on postoperative days 1, 3, and 7. Data were expressed as mean ± SD, For figure S5A, * $0.01 < P < 0.05$, ** $P < 0.01$, versus the Con group. For figure S5J, * $0.01 < P < 0.05$, ** $P < 0.01$, versus the Sham group. # $0.01 < P < 0.05$, $^{\#\#}$ $P < 0.01$, versus the IRI group. $^{\&}$ $0.01 < P < 0.05$, $^{\&\&}$ $P < 0.01$, versus the Exo group. n = 3.

ADSCs-Exo alleviates the inflammatory response following LIRI

Results of serum inflammatory cytokine detection showed that at 1 day postoperatively, compared with the IRI group, the serum levels of IL-1β, IL-6, and TNF-α in the Exo, CI, and CE groups were significantly decreased (Figure S6A-C, 0.01

< $p < 0.05$). At 3 days postoperatively, compared with the IRI group, the serum IL-1β levels in the Exo, CI, and CE groups were extremely significantly decreased (Figure S6A, $p < 0.01$); the serum IL-6 levels in the Exo and CI groups were significantly decreased (Figure S6B, $0.01 < p < 0.05$); and the serum TNF-α levels in the CE group were extremely significantly decreased (Figure S6C, $0.01 < p < 0.05$).

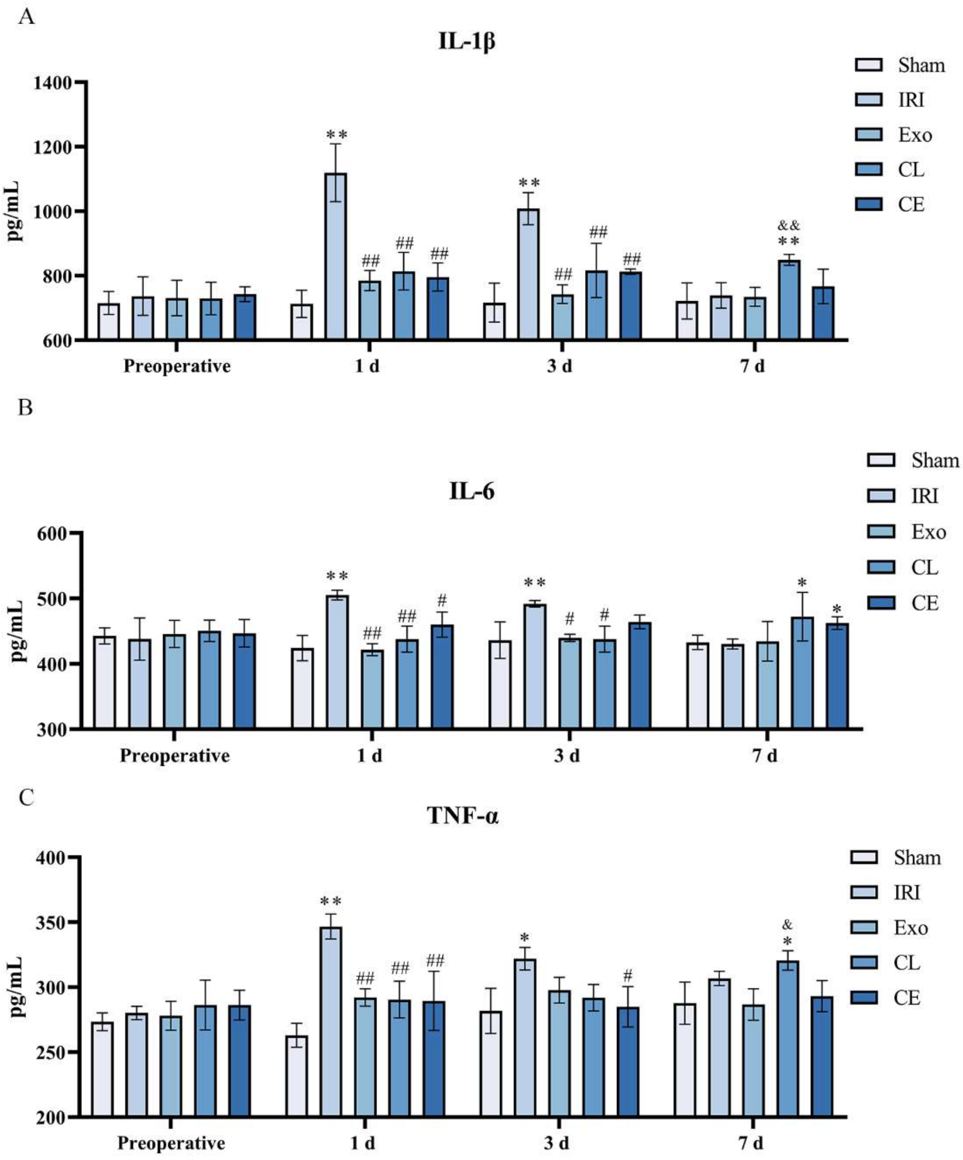

Figure S6. ADSCs-Exo alleviates the inflammatory response following LIRI. A: The IL-1β levels in the serum; B: The IL-6 levels in the serum; C: The TNF-α levels in the serum. Data were expressed as mean ± SD. * $0.01 < P < 0.05$, ** $P <$

0.01, versus the Con group. For figure S5J, * 0.01 < P < 0.05, ** P < 0.01, versus the Sham group. # 0.01 < P < 0.05, ## P < 0.01, versus the IRI group. & 0.01 < P < 0.05, && P < 0.01, versus the Exo group. n = 3.

Measurement of serum liver injury markers showed that on postoperative days 1 and 3, ALT and AST levels in the Exo, CI, and CE groups were significantly lower than those in the IRI group (Figure S7A, B; $p < 0.01$). On postoperative day 1, ALT levels in the CI and CE groups were significantly lower than those in the Exo group (Figure S7A; $0.01 < p < 0.05$). Similarly, LDH levels were significantly reduced in the Exo, CI, and CE groups compared with the IRI group (Figure S7C; $p < 0.01$), and on day 1, LDH levels in the CI and CE groups were significantly lower than those in the Exo group (Figure S7C; $p < 0.01$).

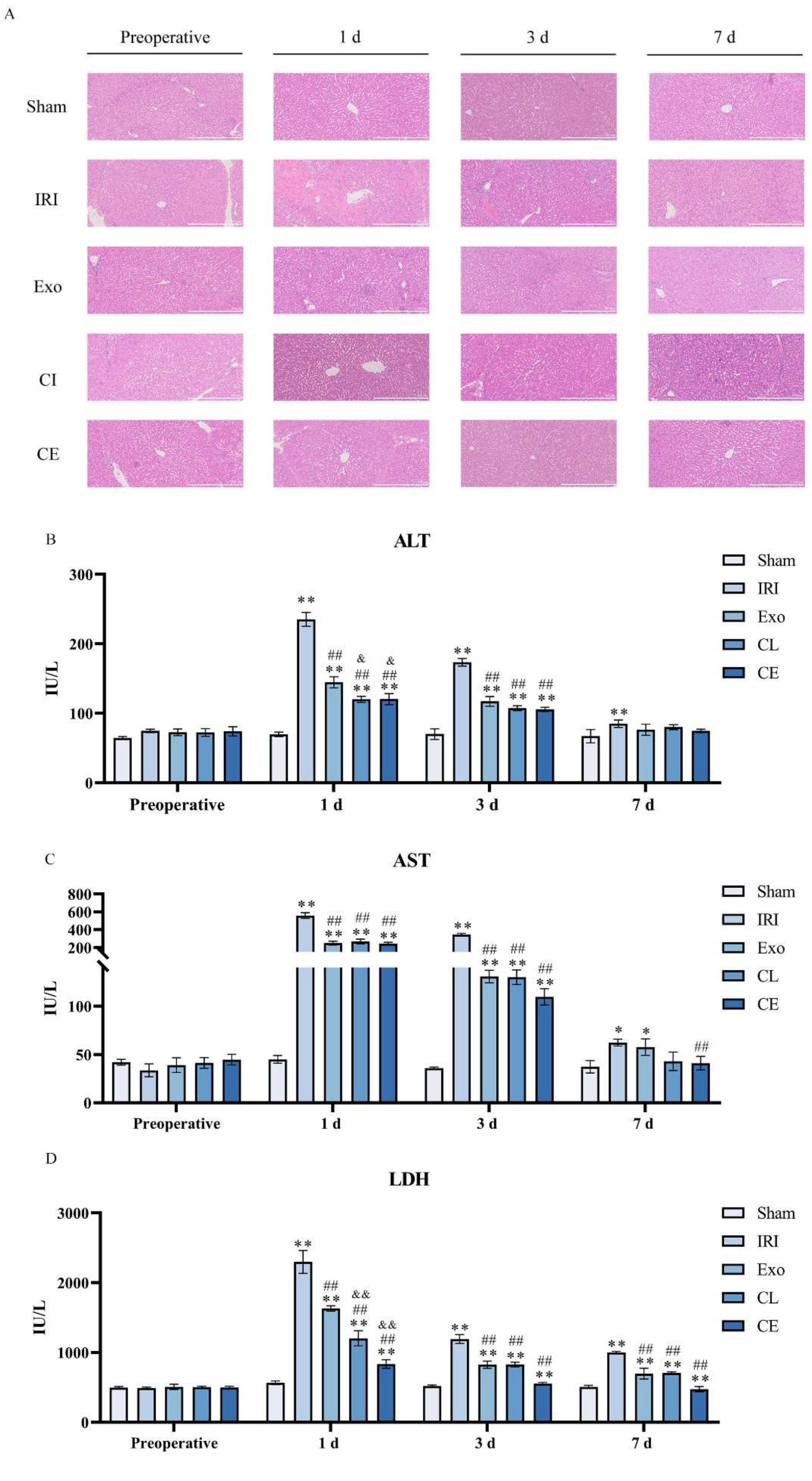

Figure S7. ADSCs-Exo alleviates liver injury following LIRI.

A: Results of HE staining (scale bar = 200 μm); B: The ALT levels in the l serum; C:

The AST levels in the serum; D: The LDH levels in the serum. Data were expressed as mean ± SD. * 0.01 < P < 0.05, ** P < 0.01, versus the Con group. For figure S5J, * 0.01 < P < 0.05, ** P < 0.01, versus the Sham group. # 0.01 < P < 0.05, $^{\#\#}$ P < 0.01, versus the IRI group. $^{\&}$ 0.01 < P < 0.05, $^{\&\&}$ P < 0.01, versus the Exo group. n = 3.
